# RFX Transcription Factor DAF-19 Regulates 5-HT and Innate Immune Responses to Pathogenic Bacteria in *Caenorhabditis elegans*

**DOI:** 10.1371/journal.pgen.1003324

**Published:** 2013-03-07

**Authors:** Yusu Xie, Mustapha Moussaif, Sunju Choi, Lu Xu, Ji Ying Sze

**Affiliations:** Department of Molecular Pharmacology, Albert Einstein College of Medicine, Bronx, New York, United States of America; University of California San Francisco, United States of America

## Abstract

In *Caenorhabditis elegans* the Toll-interleukin receptor domain adaptor protein TIR-1 via a conserved mitogen-activated protein kinase (MAPK) signaling cascade induces innate immunity and upregulates serotonin (5-HT) biosynthesis gene *tph-1* in a pair of ADF chemosensory neurons in response to infection. Here, we identify transcription factors downstream of the TIR-1 signaling pathway. We show that common transcription factors control the innate immunity and 5-HT biosynthesis. We demonstrate that a cysteine to tyrosine substitution in an ARM motif of the HEAT/Arm repeat region of the TIR-1 protein confers TIR-1 hyperactivation, leading to constitutive *tph-1* upregulation in the ADF neurons, increased expression of intestinal antimicrobial genes, and enhanced resistance to killing by the human opportunistic pathogen *Pseudomonas aeruginosa* PA14. A forward genetic screen for suppressors of the hyperactive TIR-1 led to the identification of DAF-19, an ortholog of regulatory factor X (RFX) transcription factors that are required for human adaptive immunity. We show that DAF-19 concerts with ATF-7, a member of the activating transcription factor (ATF)/cAMP response element-binding B (CREB) family of transcription factors, to regulate *tph-1* and antimicrobial genes, reminiscent of RFX-CREB interaction in human immune cells. *daf-19* mutants display heightened susceptibility to killing by PA14. Remarkably, whereas the TIR-1-MAPK-DAF-19/ATF-7 pathway in the intestinal immunity is regulated by DKF-2/protein kinase D, we found that the regulation of *tph-1* expression is independent of DKF-2 but requires UNC-43/Ca^2+^/calmodulin-dependent protein kinase (CaMK) II. Our results suggest that pathogenic cues trigger a common core-signaling pathway via tissue-specific mechanisms and demonstrate a novel role for RFX factors in neuronal and innate immune responses to infection.

## Author Summary

Toll-interleukin receptor (TIR)–domain adaptor proteins are keys to activate signaling cascades inducing transcriptional responses to internal and external pathogenic signals in evolutionary disparate organisms. Despite lacking a homolog of the mammalian innate immunity transcriptional regulator nuclear factor-kappaB (NF-κB), the nematode *Caenorhabditis elegans* responds to infections by activating TIR-1 signaling targets in the innate immune system and in neurons. Through a genetic screen for factors required for TIR-1 signaling to upregulate the serotonin biosynthesis gene *tph-1*, we identified DAF-19, an ortholog of regulatory factor X (RFX) transcription factors that were initially discovered in human immune cells. We show that DAF-19 concerts with ATF-7, a member of the activating transcription factor (ATF)/cAMP response element-binding B (CREB) family of transcription factors, to upregulate *tph-1* in the ADF chemosensory neurons and antimicrobial genes in the intestine in response to bacterial infection, reminiscent of RFX-CREB interaction in human immune cells. *daf-19* mutants display heightened susceptibility to killing by the human pathogen *Pseudomonas aeruginosa* PA14. Our studies suggest that RFX transcriptional regulation, which is essential for human adaptive immunity, has an ancient role in controlling serotonin biosynthesis and innate immunity.

## Introduction

Innate immunity is an integral part of the stress response program in which the host activates a range of defense genes to enhance the chance of survival against internal and environmental threats. In mammals, signals associated with pathogenic microbes trigger Toll-like receptors to recruit Toll-interleukin receptor (TIR) domain adaptor proteins, thereby forming scaffolds with downstream signaling cascades leading to transcriptional upregulation of defense genes. A growing body of evidence indicates that classical immune proteins including Toll-like receptors and TIR domain adaptor proteins are expressed in the developing and mature brain in mammals [1], [2]. It has been proposed that certain common molecular mechanisms may function in neurons and non-neuronal tissues to induce physiologically distinct responses to aversive cues [1], [2], [3]. Except a few cases, the gene targets of immune factors in neurons are not known and it is unclear whether those immune signaling cascades are differentially regulated in neurons and non-neuronal tissues. Consequently, identification of upstream regulators and downstream effectors of conserved core immune signaling pathways may provide insights into the regulation of the immunity as well as the regulation of neural plasticity.

Our laboratory has focused on genetic dissection of environment-dependent transcriptional regulation of the *tph-1* gene, encoding the rate-limiting serotonin (5-HT) biosynthesis enzyme tryptophan hydroxylase, in the nematode *Caenorhabditis elegans*. Previously, we showed that *tph-1* expression in a pair of ADF chemosensory neurons in the head sensory organ Amphid is modulated by two layers of transcriptional regulation according to growth conditions: signaling through the OCR-2/OSM-9 TRPV channel turns on the basal *tph-1* expression under optimal growth conditions, and aversive growth conditions further upregulate *tph-1* expression independently of OCR-2/OSM-9 [4], [5]. Work from several laboratories suggests that *tph-1* expression in the ADF neurons responds to pathogenic food. *C. elegans* feeds on bacteria and is killed by a large number of pathogenic microbes in its natural environment [6]. In an elegant study, it showed that feeding worms with the human opportunistic pathogen *Pseudomonas aeruginosa* PA14 triggers upregulation of *tph-1* in the ADF neurons leading to aversive learning and avoidance behavior [7]. A subsequent study indicated that the TIR-domain adaptor protein TIR-1, which was initially identified as an upstream regulator of a conserved mitogen-activated protein kinase (MAPK) signaling pathway in the innate immunity [8], is required for PA14-induced *tph-1* upregulation and PA14 avoidance behavior [9]. However, the *C. elegans* genome lacks a homolog of nuclear factor-kappaB (NF-κB), the major transcriptional activator of the mammalian innate immunity [10]. In addition, deletion of the sole *C. elegans* Toll receptor gene *tol-1* did not affect the intestinal immunity [11] or *tph-1* expression [7]. These observations suggest that the TIR-1 signaling cascade may involve evolutionarily more ancient upstream players and downstream transcription factors.

Activating transcription factor (RFX) transcription factors were first identified in human subjects of bare lymphocyte syndrome, a hereditary immunodeficiency disease, and are required for the expression of the major histocompatibility complex class II (MHC II) genes [12]. RFX proteins bind to the X-box motif on the MHC II promoters and interact with cAMP response element-binding (CREB) protein and other cofactors to form a higher order “enhanceosome”, which then recruits the non-DNA-binding transcriptional activator CIITA to turn on MHC II expression [13]. RFX factors have since been identified in diverse eukaryotic species [14], [15], [16] and are expressed broadly in neuronal and non-neuronal cells in animals, suggesting additional roles for RFX factors in biological processes of multiple tissues. Studies of the sole *C. elegans* RFX factor *daf-19* have uncovered its role in the development of dendritic cilia of sensory neurons [17], [18]. Subsequent studies found RFX factors regulating ciliogenesis in *Drosophila* [19] and mouse [20], demonstrating one aspect of RFX function conserved across phyla.

In this paper, we identified DAF-19 as a key transcriptional regulator of *tph-1* in the ADF neurons and intestinal antimicrobial genes in *C. elegans*. We found that, analogous to the RFX-CREB interaction for MHC II expression in human immune cells, DAF-19 concerts with ATF-7, a member of activating transcription factor (ATF)/CREB superfamily of transcription factors, acting downstream of the TIR-1 signaling cascade to control transcriptional responses to pathogenic bacterial food in *C. elegans*. We show that the TIR-1-DAF-19/ATF-7 pathway is differentially regulated to induce *tph-1* upregulation and intestinal immunity in response to *P. aeruginosa* PA14. Thus, our data suggest that pathogenic signals may trigger a common core signaling pathway via cell-specific mechanisms and a RFX transcription factor acts in an ancient host to regulate 5-HT biosynthesis and the innate immunity.

## Results

### Isolation of *tir-1(yz68)* gain-of-function mutation

We carried out a forward genetic screen to identify components underling aversive environment-induced *tph-1* upregulation in *C. elegans*. The levels of *tph-1* expression in identified neurons in living *C. elegans* can be estimated by quantifying fluorescence intensity of a green fluorescence protein (GFP) driven by the *tph-1* promoter (*tph-1::gfp*) [4]. A pair of ADF neurons is the only chemosensory neurons producing 5-HT in a hermaphrodite *C. elegans* [21]. Each ADF neuron projects a single dendrite to the tip of the nose where the ciliated sensory endings are exposed to the external environment and its axon extends to the nerve ring, the brain of *C. elegans* [22]. We started with a strain expressing a stably chromosomally integrated *tph-1::gfp* transgene in *ocr-2(yz5)* mutant background, in which *tph-1::gfp* expression in the ADF neurons is visible under aversive growth conditions but not under optimal growth conditions, providing a visual assay for environment-dependent changes in *tph-1* expression [5]. We isolated mutagenized worms with enhanced ADF *tph-1::gfp* under optimal growth conditions, and analyzed the mutants in the *ocr-2* background as well as after the *ocr-2* mutation being outcrossed. *yz68* is one of the mutants retrieved from the screen.

**Figure 1:**
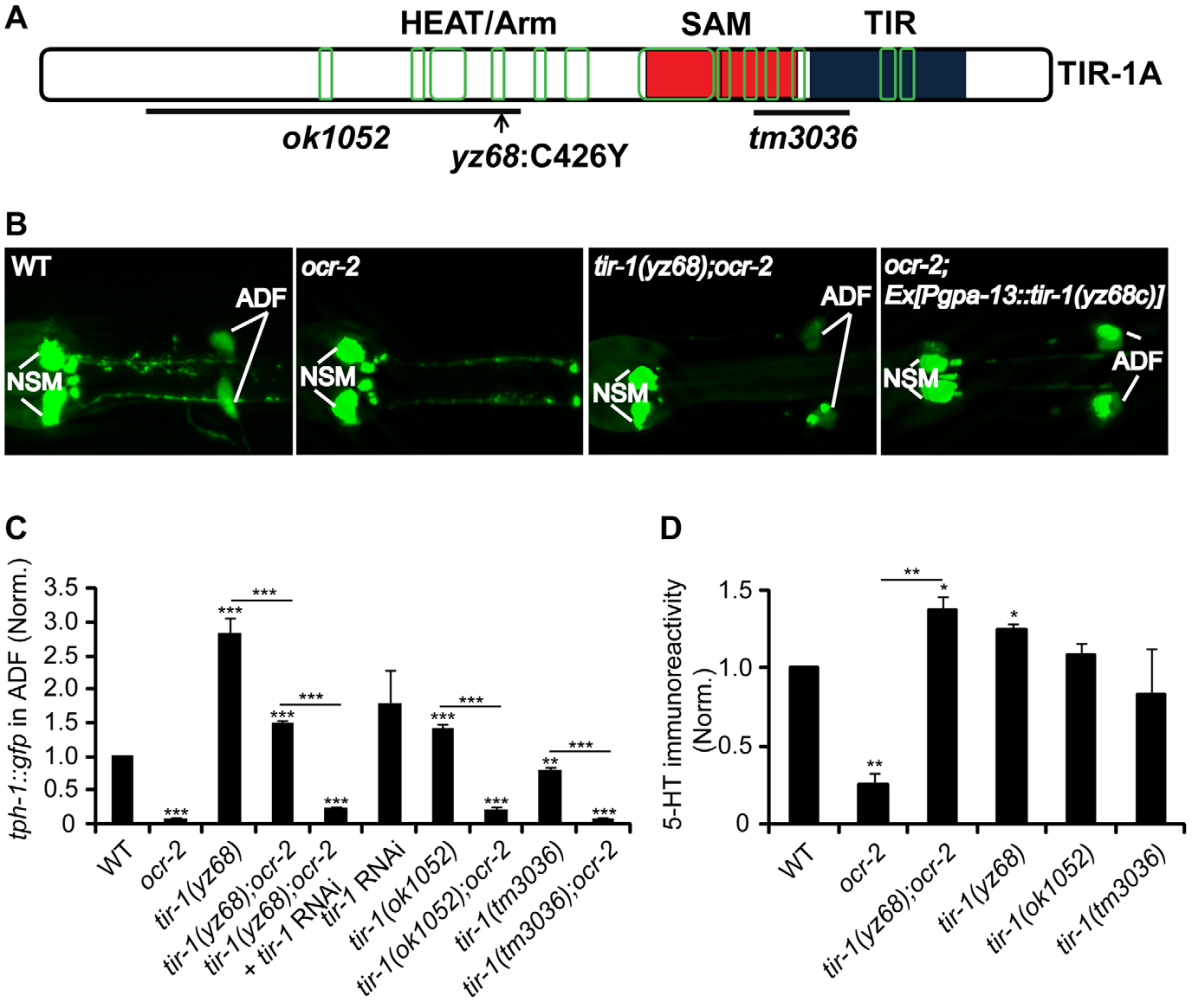
*tir-1(yz68gf)* mutants constitutively upregulate *tph-1::gfp* expression in ADF neurons. A. Mutations in predicted *tir-1* protein. The TIR-1 structure is adapted from a previous report [8]. Blue, red and green denote the TIR domain, SAM domains, and regions containing the HEAT/Arm motifs, respectively. B. Photomicrographs showing that *tir-1(yz68gf)* mutation and transgenic expression of *tir-1(yz68gf)* cDNA enhanced ADF *tph-1::gfp* expression. *ocr-2* background was used to reduce basal ADF *tph-1::gfp* for a better detection of *tph-1::gfp* upregulation by *tir-1(yz68gf)*. C. Quantification of ADF *tph-1::gfp* fluorescence in *tir-1(lf) and tir-1(gf)* mutants under optimal growth conditions. *tir-1(yz68gf)* mutants displayed enhanced ADF fluorescence on its own and in *ocr-2* backgrounds relative to corresponding controls, and RNAi of *tir-1* abolished *tph-1::gfp* upregulation by *tir-1(yz68gf)*. The value of GFP fluorescence in the ADF neurons of mutants is normalized to that of WT animals. For RNAi experiments, the value of *tir-1* RNAi is normalized to that in the same strain but treated with an empty vector. Each bar represents the average of at least three trials ± SEM. Throughout of this paper, statistics between WT and individual mutants is marked on the top of each bar, and that between two mutant strains or two treatments is indicated on the top of the line across compared strains, *** p<0.001. D. Quantification of 5-HT immunoreactivity in ADF neurons of *tir-1* mutants. Data represent the average of two trials, each with at least 20 L4-stage animals per strain ± SEM, and the value of the mutants is normalized to that of WT animals stained in parallel, *p<0.05, ** p<0.01.

Through single nucleotide polymorphism-based (SNP) mapping, RNA-interference (RNAi)-mediated inactivation of candidate genes in the mapped contig, and sequencing the *yz68* mutant genome, we identified a nucleic acid change predicting a substitution of cysteine_426_ by tyrosine (C426Y) in the fourth ARM motif of the HEAT/Arm repeat region of the TIR-1 protein (Figure 1A). Several experimental data suggest that the C426Y substitution causes TIR-1 constitutive activation. First, whole mount anti-5-HT-antibody staining showed that ADF 5-HT immunoreactivity in *tir-1(yz68);ocr-2* double mutants was elevated compared to the *ocr-2* single mutant (Figure 1D). As 5-HT is being secreted, 5-HT immunostaining does not fully reflect the rate of 5-HT biosynthesis. With this caveat in mind, the results suggest that *tir-1(yz68)* enhanced 5-HT in the ADF neurons. Second, transgenic expression of *tir-1(yz68)* cDNA under the *gpa-13* promoter (*Pgpa-13::tir-1(yz68c*)), which is expressed in ADF, AWC and ASH sensory neurons in the head region, recapitulated *tph-1::gfp* upregulation (Figure 1B). Third, RNAi of *tir-1* in *tir-1(yz68);ocr-2* mutants blocked the *tph-1* upregulation (Figure 1C). The *tir-1(ok1052)* mutation, which causes mixed gain- and loss-of-function *tir-1* phenotypes in the AWC neuron development [23] but does not affect the innate immunity [24], caused only a modest increase in ADF *tph-1::gfp* (Figure 1C). Collectively, these data suggest that the C426Y substitution alters a site critical for TIR-1 activation in the ADF neurons.

Unlike the *ocr-2* mutation, *tir-1(tm3036)* loss-of-function (lf) mutants and RNAi of *tir-1* do not lead to a dramatic reduction in ADF *tph-1::gfp* (Figure 1C). Likewise, inactivation of TIR-1 downstream MAPKKK *nsy-1* or MAPKK *sek-1* by RNAi and deletion, respectively, did not downregulate *tph-1::gfp*, although RNAi of *nsy-1* did abolish *tph-1::gfp* upregulation by *tir-1(yz68gf)* (Figure S1). These observations are consistent with a published work [9]. These data suggest that TIR-1 signaling is not a major regulator of basal *tph-1* expression in the ADF neurons under optimal growth conditions.

### TIR-1 signaling pathway selectively regulates serotonergic response to pathogenic food

**Figure 2:**
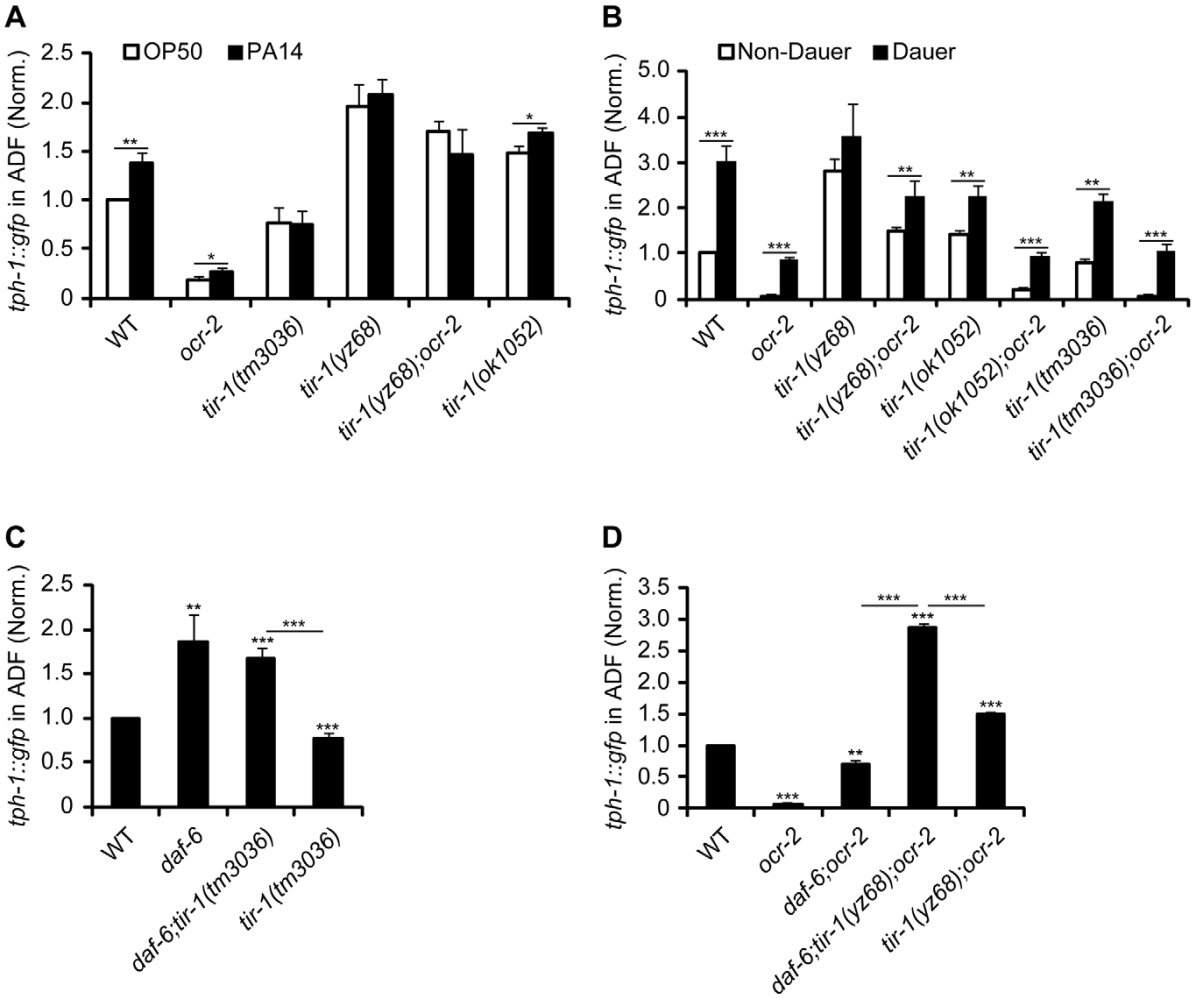
TIR-1 signaling selectively regulates *tph-1::gfp* response to pathogenic bacterial food. A. PA14-induced ADF *tph-1::gfp* upregulation was impaired in *tir-1(tm3036lf)* and *tir-1(yz68gf)* mutants. B. *tir-1(tm3036lf)* and *tir-1(yz68gf)* mutations did not block *tph-1::gfp* upregulation during dauer formation. C. TIR-1 is not required for *tph-1::gfp* upregulation caused by changes in cilial morphology. Mutations in *daf-6*/Patched alter the morphology of dendritic cilia of all chemosensory neurons including ADF and cause ADF *tph-1::gfp* upregulation on its own as well as in *tir-1(lf)* background. D. *tir-1(yz68gf)* and *daf-6* mutations confer additive upregulation of *tph-1::gfp* in the ADF neurons. Data represent the average of three trials each with at least 20 animals per strain per condition ± SEM. The value of ADF GFP fluorescence in WT animals under a stress paradigm and that of mutants is normalized to the value of WT animals under optimal conditions. *p<0.05, ** p<0.01, *** p<0.001.

Previously, we identified that a number of aversive conditions may induce *tph-1* upregulation in the ADF neurons [5]. We asked whether *tir-1* signaling specifically mediates the response to pathogenic bacteria, or it is responsible for *tph-1* upregulation under all aversive conditions. We first analyzed the intensity of *tph-1::gfp* in the ADF neurons in *tir-1(lf) and tir-1(gf)* mutants fed with the pathogenic *P. aeruginosa* strain PA14. Following feeding on PA14 for 6 hr, ADF fluorescence in WT animals was ∼1.4-fold higher than their sibling on OP50, but *tir-1(lf)* mutants failed to upregulate *tph-1::gfp* under the same conditions (Figure 2A), consistent with prior reports [7], [9]. The *tir-1(yz68gf)* mutant also did not exhibit a significant increase in *tph-1::gfp* following PA14 feeding, suggesting that the pathogen signals cannot further enhance *tir-1(yz68gf)* protein activity (Figure 2A).

We next tested if TIR-1 function is required for *tph-1* upregulation during dauer formation. Under the conditions of starvation, high growth temperature and high levels of pheromones, *C. elegans* develops into a stress-resistant dauer larva through a series of cellular and physiological remodeling and turns on a battery of stress genes [25]. We previously showed that WT and *ocr-2* mutant worms upregulated ADF *tph-1::gfp* when they entered the dauer stage [5]. We therefore induced *tir-1(lf)* and *tir-1(gf)* mutants to form dauers by treating the worms with dauer pheromones. We observed ADF *tph-1::gfp* upregulation in both *tir-1(tm3036lf)* and *tir-1(tm3036lf);ocr-2* double mutant dauers as compared with corresponding L4-stage animals (Figure 2B). ADF *tph-1::gfp* was increased in *tir-1(yz68gf)* mutants during dauer formation and this increase was more evident in the *tir-1(yz68gf);ocr-2* double mutant dauers (Figure 2B). Thus neither TIR-1 deficiency nor TIR-1 hyperactivation can block *tph-1::gfp* upregulation induced by dauer formation.

We previously showed that mutations that alter the morphology of dendritic cilia of ADF neurons cause *tph-1* upregulation [5]. We therefore tested whether aberrant cilia trigger TIR-1 leading to *tph-1::gfp* upregulation. If this were the case, then we can expect that inactivation of *tir-1* blocks *tph-1::gfp* upregulation in cilial mutants. Mutations of *daf-6*/Patched, which is expressed in the glia ensheathing the dendritic cilia of the chemosensory neurons [26], and the Intraflagellar Transport (IFT) gene *dyf-1* that is essential for cilia formation [27] conferred ADF *tph-1::gfp* upregulation [5]; however, inactivation of *tir-1* did not block *tph-1::gfp* upregulation in *daf-6* (Figure 2C) or *dyf-1* mutants (Figure S2). Furthermore, a triple mutant of *daf-6*, *tir-1(yz68gf);ocr-2* showed higher ADF *tph-1::gfp* than *daf-6;ocr-2* and *tir-1(yz68gf);ocr-2* double mutants (Figure 2D). Together these data suggest that the ADF neurons can detect and discriminate multiple aversive cues, and indicate that TIR-1 is selectively involved in the pathogen signaling transduction pathway.

### *daf-19* is a suppressor of *tir-1(yz68gf)*

**Figure 3:**
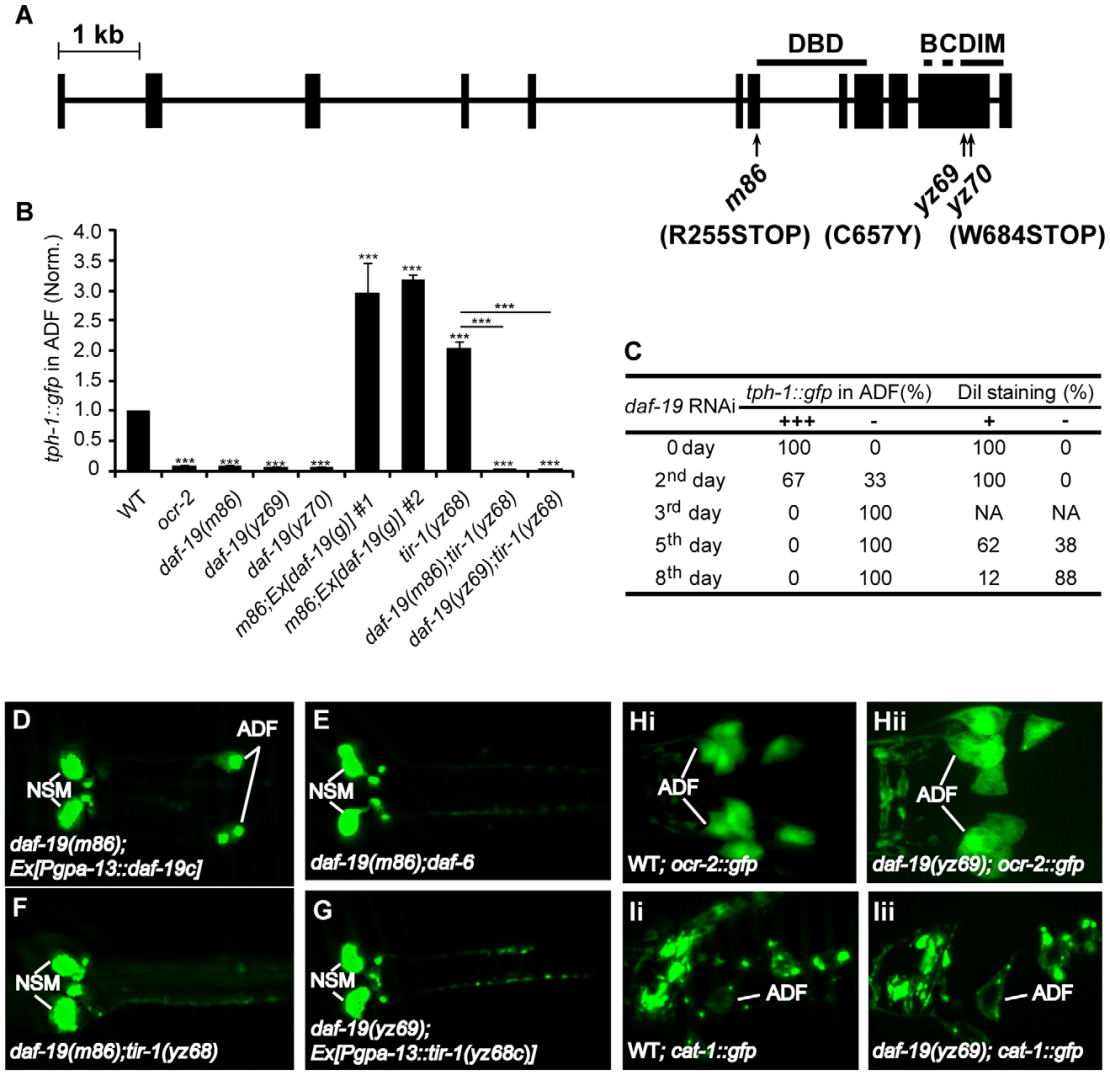
*daf-19* is required for *tph-1* expression in the ADF neurons. A. Mutations in the *daf-19* gene. The *daf-19* gene structure is adapted from a previous report [17]. Boxes denote exons and the line denotes introns. The areas containing the DNA binding domain (DBD), dimerization domain (DIM) and conserved B and C regions are indicated. B. ADF *tph-1::gfp* is dramatically reduced in *daf-19* mutants and *daf-19;tir-1(yz68gf)* double mutants. Two transgenic lines expressing WT *daf-19* genomic sequence rescued *tph-1::gfp* expression in *daf-19(m86)*-null mutants. *** p<0.001. C. RNAi of *daf-19* after embryogenesis. L1 larvae with normal ADF *tph-1::gfp* and cilial morphology were fed *E. coli* expressing RNAi clone against *daf-19*. *tph-1::gfp* was quantified on indicated days, and dye filling of chemosensory neurons with fluorescence dye DiI was performed to monitor gross cilia morphology of the chemosensory neurons. *daf-19* RNAi eliminated ADF *tph-1::gfp* (-) and eliminated dye filling of the cilia, but *tph-1::gfp* was abolished in many animals prior to a detectable change in the cilia morphology. -, No discernible fluorescence of *tph-1::gfp* or DiI staining in the neurons. D–G. Photomicrographs showing *tph-1::gfp* in *daf-19* mutants under various genetic backgrounds. *daf-19* deficiency abolished *tph-1::gfp* expression in the ADF neurons of both *daf-6* and *tir-1(yz68gf)* backgrounds. H–I. Expressions of ADF marker genes in *daf-19* mutants. *ocr-2::gfp* and VMAT *cat-1::gfp* were comparable between WT (Hi and Ii) and *daf-19* mutant (Hii and Iii) animals, suggesting that *daf-19* deficiency does not grossly alter ADF cell fates.

To identify the effectors of TIR-1 signaling, we carried out a suppressor screen for mutants that abrogate *tph-1* expression in *tir-1(yz68gf)* mutants. Using a combination of SNP mapping, non-complementation tests and sequencing the mutant genomes, we identified that two mutations, *yz69* and *yz70*, are allelic to the *daf-19* gene, encoding the sole *C. elegans* ortholog of the RFX transcription factors (Figure 3A). Subsequent experiments with our alleles and the previously existing *daf-19(m86)*-null mutation revealed that DAF-19 function is required not only for *tir-1(yz68gf)* to upregulate *tph-1::gfp*, but also for ADF *tph-1::gfp* expression under optimal growth conditions (Figure 3B), during dauer formation (data not shown) and in aberrant cilia backgrounds (Figure 3E; Figure S2). The reduced *tph-1::gfp* in *daf-19* mutants was fully rescued by transgenic expression of WT *daf-19* genomic sequence (Figure 3B) or *daf-19* cDNA driven by the *gpa-13* promoter (Figure 3D).

An implicit concern was that *daf-19* deficiency alters ADF cell fates. To directly analyze the effect of *daf-19* deficiency on *tph-1* expression, we inactivated *daf-19* by RNAi after 5-HT phenotypes established. Figure 3C shows that 100% of larval stage 1 (L1) worms expressed *tph-1::gfp* prior to RNAi treatment, 33% of the animals lost ADF GFP after 24 hr RNAi treatment, and by 48 hr, 100% of the animals showed no GFP in the ADF neurons. DAF-19 is required for the expression of cilia IFT components [17]. Although we showed that aberrant cilia induced ADF *tph-1::gfp* upregulation [5] (Figure S2), complete lacking cilia could inhibit *tph-1* expression. To rule out this possibility, we used lipophilic dye DiI staining to examine the dendritic cilia morphology of the chemosensory neurons in worms treated with *daf-19* RNAi. We observed that RNAi of *daf-19* eliminated ADF *tph-1::gfp* prior to a detectable change in the cilia (Figure 3C).

We investigated whether the reduced *tph-1* expression is a secondary consequence of reduced *ocr-2* and *tir-1* expression in *daf-19* mutants. *daf-19(yz69)* (Figure 3Hii) and *daf-19(m86)*-null (not shown) mutants expressed a GFP reporter for *ocr-2* (*ocr-2::gfp*) in ADF and other chemosensory neurons indistinguishable from WT animals. Diminished *tph-1::gfp* expression also cannot be ascribed to reduced *tir-1* expression, as transgenic expressing *tir-1(yz68)* cDNA by the *gpa-13* promoter failed to increase ADF *tph-1::gfp* in *daf-19* mutants (Figure 3G), although the *Pgpa-13::daf-19c* transgene did (Figure 3D), indicating that the *gpa-13* promoter is expressed in *daf-19* mutants but that the *tir-1(yz68gf)* protein cannot stimulate *tph-1* expression in the absence of DAF-19 function.

To assess whether *daf-19* deficiency abolishes all 5-HT phenotype genes, we analyzed the expression of *cat-1*, encoding the vesicular monoamine transporter required for 5-HT synaptic release [28]. GFP-tagged CAT-1 was expressed and localized properly in the ADF neurons of *daf-19* mutants (Figure 3Iii). Thus, diminished *tph-1::gfp* in *daf-19* mutants cannot be attributed to altered ADF cell fate.

### TIR-1-induced *tph-1* upregulation requires DAF-19 and ATF-7

**Figure 4:**
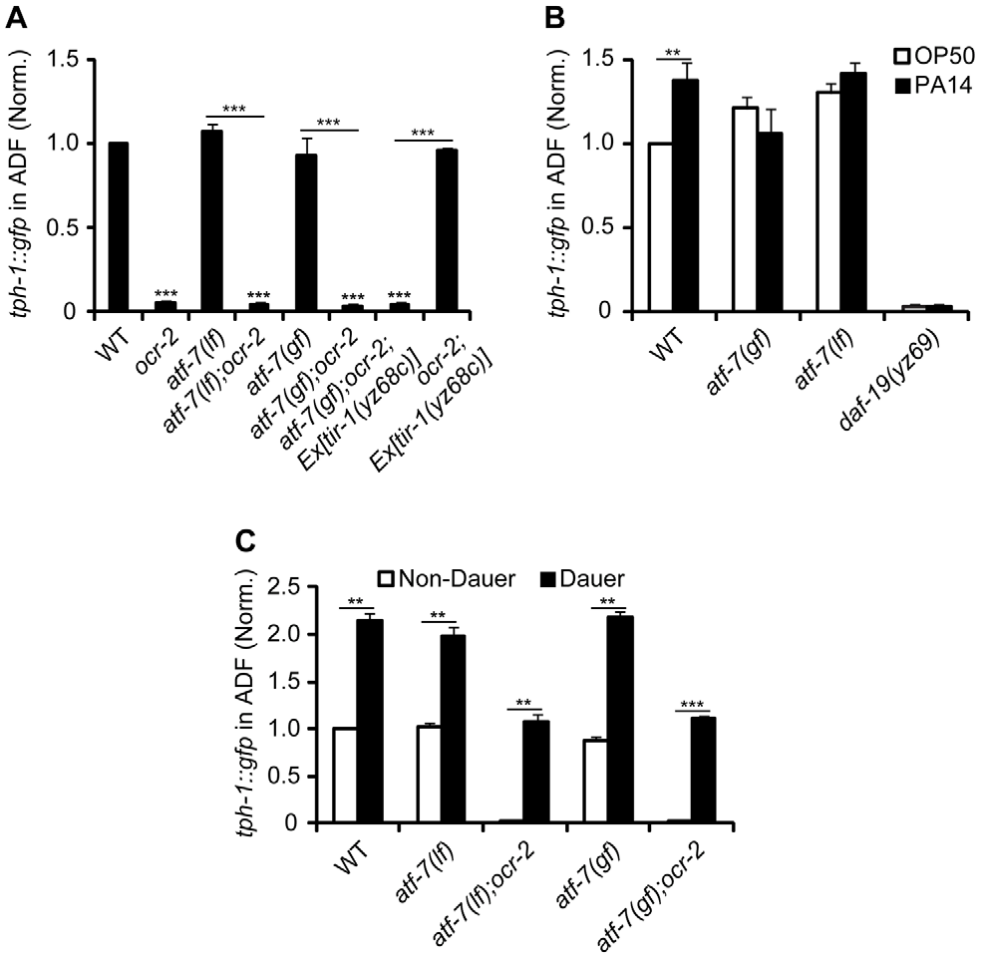
Constitutive repressor function of *atf-7(gf)* mutation blocks *tph-1::gfp* upregulation induced by *tir-1(gf)* and PA14. A. Quantification of ADF *tph-1::gfp* in *atf-7(lf)*, *atf-7(gf)* and *atf-7(gf)* carrying the *Ex[tir-1(yz68gf)]* transgene under optimal growth conditions, on their own or in the background of *ocr-2* mutation. B. *atf-7(lf)*, *atf-7(gf)* and *daf-19* mutants failed to upregulate ADF *tph-1::gfp* following 6 hr incubation on a lawn of PA14 relative to their sibling incubated on OP50. One-day-old adults were tested. C. *atf-7(lf)* and *atf-7(gf)* mutations, on their own or in *ocr-2* mutation background, did not prevent ADF *tph-1::gfp* upregulation during dauer formation, as compared to L4 worms. Data represent the average of three trials each with at least 20 animals per strain per condition ± SEM. The value of GFP fluorescence in the ADF neurons of WT animals under a stress paradigm and that of mutants is normalized to the value of WT animals under optimal conditions. ** p<0.01, *** p<0.001.

Because *daf-19* appeared to be required for *tph-1* expression controlled by multiple mechanisms, we hypothesized that DAF-19 may concerts with other transcriptional regulators to confer specificity to particular signaling pathway activation. In light of the partnership of RFX and CREB proteins for MHC II expression, we further hypothesized that an analogous mechanism might underscore transcriptional responses to pathogenic signals in *C. elegans*. ATF-7, an ortholog of the mammalian ATF2/ATF7/CREB5 family of transcription factors [29], has been implicated as a transcriptional repressor of antimicrobial genes in the intestine; activation of PMK-1 p38 MAPK de-represses ATF-7, hence upregulating the antimicrobial genes [30]. We therefore analyzed *tph-1::gfp* expression in *atf-7(gf)* and *atf-7(lf)* mutants. *atf-7(gf)* mutation presumably constitutively represses TIR-1 signaling targets. *atf-7(gf)* did not downregulate ADF *tph-1::gfp* under optimal growth conditions (Figure 4A), as seen in mutants with reduced *tir-1*, TIR-1 downstream *nsy-1*/MAPKKK and *sek-1*/MAPKK (Figure 1C, Figure S1) [9]. Like *tir-1(lf)* mutants, *atf-7(gf)*, as well as *daf-19* mutants, failed to upregulate ADF *tph-1::gfp* following 6 hr feeding on PA14 (Figure 2A, Figure 4B). By contrast, both *atf-7(gf)* and *atf-7(lf)* mutants upregulated ADF *tph-1::gfp* during dauer formation (Figure 4C). These data suggest that ATF-7 confers the specificity to upregulate *tph-1* in response to pathogenic bacteria.

We tested further whether constitutive repression function of *atf-7(gf)* could suppress *tph-1::gfp* upregulation by *tir-1(yz68gf)*. We crossed the *Pgpa-13::tir-1(yz68c)* transgene into *atf-7(gf)* mutants. The *Pgpa-13::tir-1(yz68c)* transgene conferred a significantly increase of ADF *tph-1::gfp* as tested in *ocr-2* mutant background but not in *ocr-2;atf-7(gf)* double mutants (Figure 4A). Thus, *tph-1* upregulation induced by PA14 and *tir-1(yz68gf)* requires both DAF-19 and ATF-7.

### *daf-19* deficiency suppresses the enhanced innate immunity of *tir-1(yz68gf)* mutants

**Figure 5:**
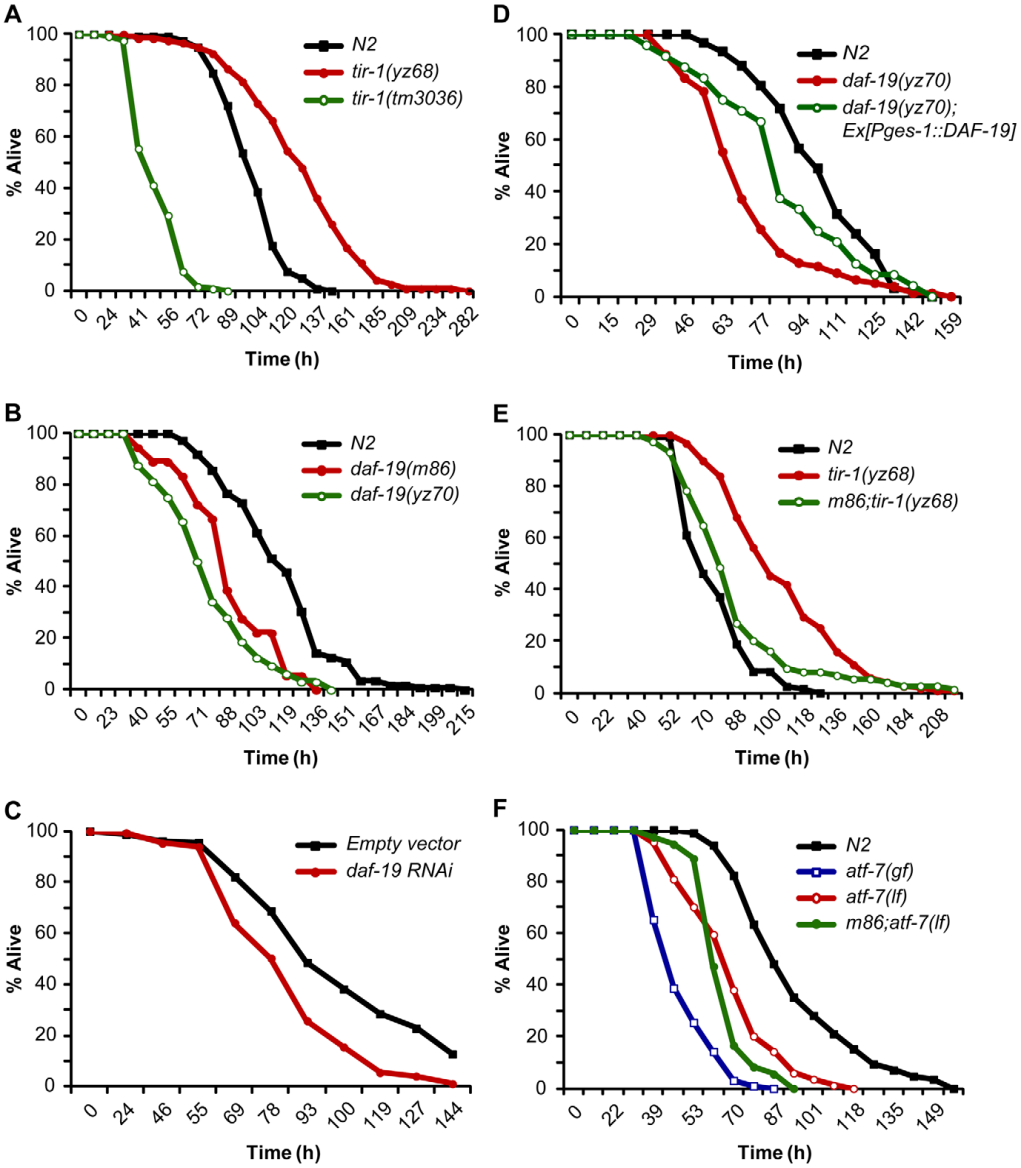
DAF-19 regulation of TIR-1-mediated innate immunity. A. Survival rates of WT and loss- and gain-of-function mutants of *tir-1* fed PA14 under standard slow killing pathogenesis assay conditions. B. *daf-19* mutants displayed elevated susceptibility to killing by PA14. C. Survival rates of vector control and *daf-19* RNAi-treated worms fed PA14. D. Transgenic expression of *daf-19* in the intestine by the *ges-1* promoter partially rescued the elevated PA14 susceptibility of *daf-19* mutants. E. *daf-19* deficiency suppressed the enhanced PA14 resistance of *tir-1(yz68gf)* mutants. F. *daf-19;atf-7(lf)* mutants displayed elevated susceptibility to killing by PA14 similarly to *atf-7(lf)* mutants. The fraction of live animals was determined at each indicated time points. The experiments were performed in triplicates. Results are representative of at least two independent experiments.

*daf-19* is expressed broadly in neurons but also in the intestine (Figure S3) [31], [32]. We used epistasis analysis to investigate the role of DAF-19 in TIR-1-mediated innate immunity. When incubated on a lawn of PA14, *tir-1(yz68gf)* mutants exhibited enhanced resistance to killing by PA14, in contrast to the heightened susceptibility of *tir-1(lf)* mutants (Figure 5A). *daf-19(m86)* and *daf-19(yz70)* mutants exhibited heightened susceptibility to PA14 compared to WT (Figure 5B). Similarly, RNAi of *daf-19* enhanced the susceptibility to PA14 compared to mock RNAi (Figure 5C). Transgenic expression of *daf-19* cDNA in the intestine partially rescued PA14 resistance in *daf-19* mutants (Figure 5D). *daf-19(m86)*-null mutation suppressed enhanced immune resistance of the *tir-1(yz68gf)* mutants, showing that the enhanced pathogen resistance of *tir-1(yz68gf)* mutants also requires DAF-19 function (Figure 5E). However, the *daf-19(m86);tir-1(yz68gf)* double mutants did not display heightened pathogen susceptibility as seen in *daf-19(m86)* single mutants, suggesting additional transcriptional regulators involved in the immunity induced by TIR-1 activation.

We tested the functional relationship between DAF-19 and ATF-7 in the innate immunity. Both *atf-7(gf)* and *atf-7(lf)* mutants exhibited heightened susceptibility to killing by PA14, although the immunodeficiency phenotype of *atf-7(lf)* mutants is weaker [30]. If ATF-7 and DAF-19 function in parallel, we could expect a stronger immunodeficiency phenotype in a double mutant of *atf-7(lf)* and *daf-19* relative to the single mutants. Contrary to the prediction, *atf-7(lf);daf-19* double mutants displayed a survival rate on PA14 comparable to the *atf-7(lf)* single mutant (Figure 5F). This result is more consistent with the model in which DAF-19 regulates ATF-7 targets in the immune system.

### TIR-1- and ATF-7-regulated immune reporter genes are also regulated by DAF-19

**Figure 6:**
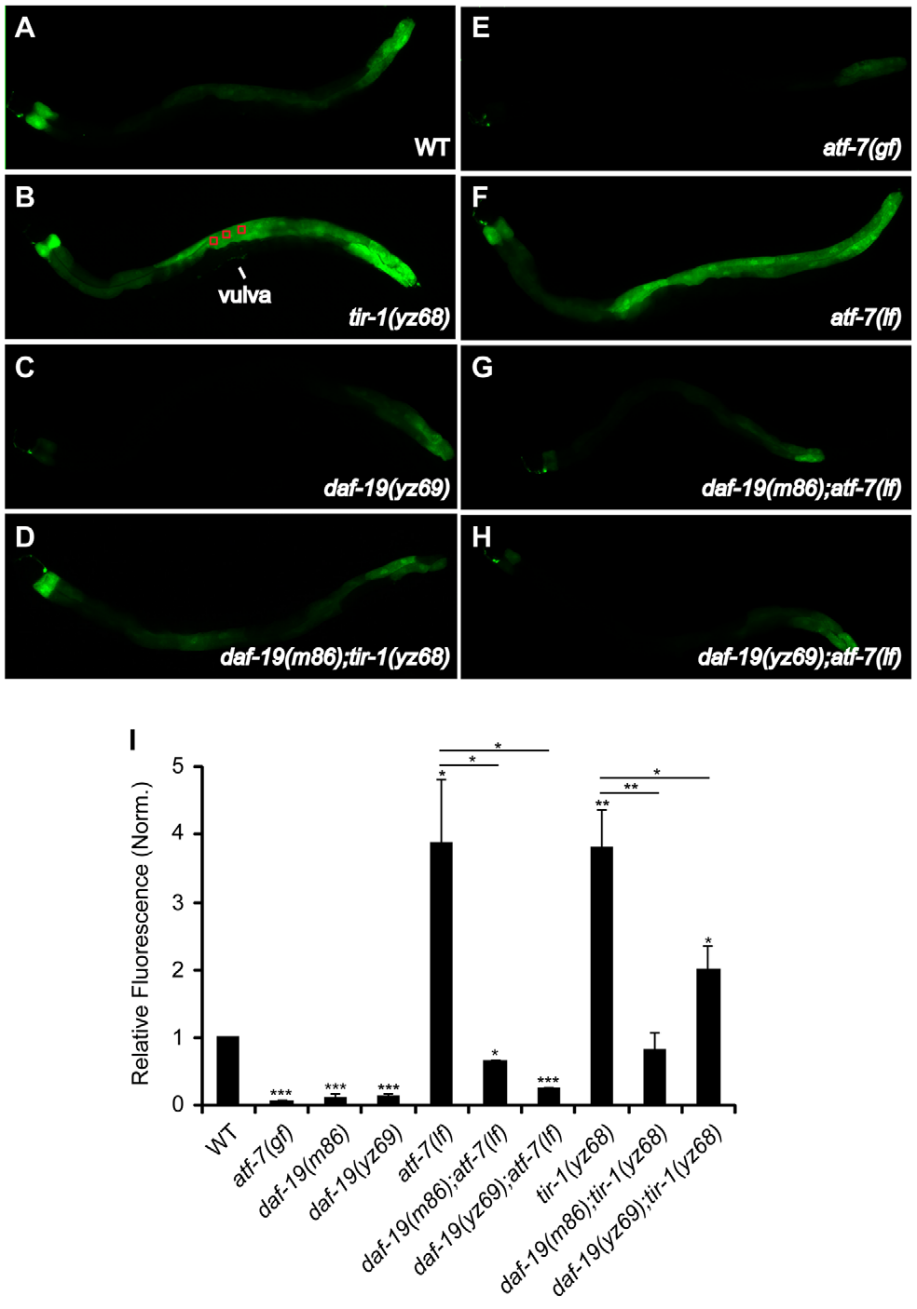
Reduced immune gene marker in the intestine of *daf-19* mutants. A–H. Representative photomicrographs showing two-day-old adult WT and indicated mutants expressing a GFP reporter for the ShK-like toxin peptide gene *T24B8.5 (T24B8.5::gfp)* in the intestine, all the animals shown with the anterior to the left. I. Quantification of *T24B8.5::gfp* intensity in the intestine of two-day-old adults. Fluorescence was quantified by measuring pixel intensity of three areas along the body (as depicted in B). *T24B8.5::gfp* intensity was reduced in *atf-7(gf)* and two *daf-19* alleles, but increased in *tir-1(yz68gf)* and *atf-7(lf)* mutants. *daf-19* deficiency diminished the increased *T24B8.5::gfp* in *tir-1(yz68gf)* as well as *atf-7(lf)* mutants. Data represent the average of three trials each with 20 animals per strain ± SEM, and the value of GFP fluorescence in mutants is normalized to that of WT animals analyzed on the same day. Statistics between WT and mutants is marked on the top of each bar, and that between two indicated groups is marked on the top of lines, * p<0.05, ** p<0.01, *** p<0.001.

The exact detoxification mechanisms of the *C. elegans* immunity are not known. To validate the role of DAF-19 in the innate immunity, we made use of the fact that bacterial infections cause intestine to induce the transcription of a battery of secretory proteins that are thought to produce antimicrobial effects [33], [34]. Transcriptional regulation of those candidate antimicrobial genes has been used as an assay for genetic delineation of *C. elegans* immune pathways [35], [36]. For example, the *atf-7(gf)* allele, as well as a number of *tir-1(lf)* alleles, were identified based on the diminished intestinal expression levels of a GFP reporter for the ShK-like toxin peptide gene T24B8.5 (*T24B8.5::gfp*), and *atf-7(lf)* alleles were identified as suppressors of the diminished *T24B8.5::gfp* of *atf-7(gf)* [30]. We therefore analyzed the same integrated *T24B8.5::gfp* reporter in *tir-1* and *daf-19* mutants. On a lawn of standard bacterial food *E. coli* OP50, *T24B8.5::gfp* intensity in the intestine of *tir-1(yz68)* was substantially enhanced relative to WT animals (Figure 6B, 6I), further validating the constitutive activity of *tir-1(yz68gf)*. By contrast, intestinal *T24B8.5::gfp* was markedly reduced in *daf-19* mutants as in *atf-7(gf)* and *tir-1(lf)* mutants (Figure 6C, 6E, 6I). Thus, DAF-19 deficiency results in downregulation of an immune gene marker regulated by TIR-1 and ATF-7.

Similar to the effect of the *daf-19* mutation on *tir-1(yz68gf)* immunity (Figure 5E), the *daf-19(m86)* and *daf-19(yz69)* mutations diminished the increased *T24B8.5::gfp* expression in *tir-1(yz68gf)* mutants, although the GFP level in the *daf-19; tir-1(yz68gf)* double mutants was higher compared to the *daf-19* single mutants (Figure 6D, 6I). We also detected an increase in *T24B8.5::gfp* in the *atf-7(lf)* intetine relative to WT animals; two tested *daf-19* alleles both reversed the increased *T24B8.5::gfp* in *atf-7(lf)* mutants (Figure 6F, 6G, 6H, 6I).

**Figure 7:**
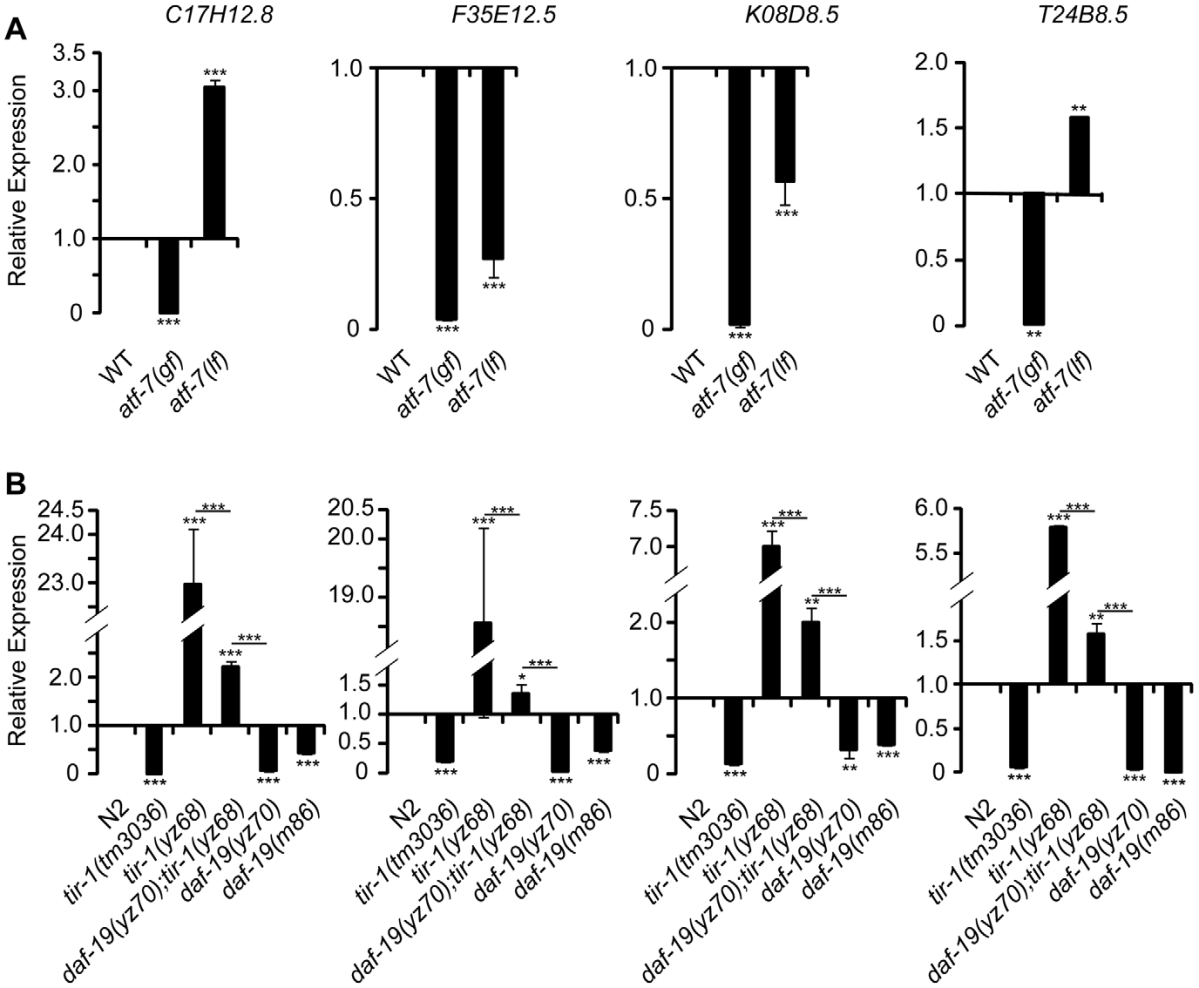
qPCR analysis of predicted antimicrobial genes in *daf-19* mutants. A. Expression levels of four predicted antimicrobial genes in adult *atf-7(gf)* and *atf-7(lf)* mutants. B. Expression levels of the predicted antimicrobial genes in adult *tir-1(lf)*, *tir-1(yz68gf)*, two *daf-19* alleles and *daf-19;tir-1(yz68gf)* double mutants. The levels of all tested genes were reduced in both *daf-19* alleles and *tir-1(lf)* alleles. *daf-19(lf)* diminished the enhanced expression of the antimicrobial genes in *tir-1(yz68gf)* mutants. For each gene, the expression level in WT is defined as 1, and that of mutants is relative to WT. Experiments were carried out in triplicates. Results are representative of two independent experiments. Analysis of WT and indicated mutants at the L4 stage showed comparable results. Statistics between WT and mutants is marked on the top of each bar, and that between two indicated groups is marked on the top of lines, ** p<0.01, *** p<0.001.

To confirm the role for DAF-19 in the regulation of candidate antimicrobial genes, we used quantitative real-time RT-PCR (qPCR) to measure the expression of endogenous *T24B8.5* as well as three other ATF-7-regulated candidate antimicrobial genes. Every tested antimicrobial gene was reduced in two tested *daf-19* alleles compared to WT animals (Figure 7B). In contrast, the message levels of these antimicrobial genes were increased in *tir-1(yz68gf)* mutants (Figure 7B). *daf-19* mutation diminished the increases of those antimicrobial genes in *tir-1(yz68gf)* mutants (Figure 7B), similar to that seen with *T24B8.5::gfp* in the intestine. As the controls, we analyzed *atf-7(lf)* and *atf-7(gf)* mutants. We observed that the message levels of *K08D8.5* and *F35E12.5* were significantly reduced, and *C17H12.8* elevated in the *atf-7(lf)* mutant (Figure 7A), as previously reported [30]. Consistent with our observation of enhanced *T24B8.5::gfp* in *atf-7(lf)* intestine, we found *T24B8.5* message level increased in *atf-7(lf)* mutants (Figure 7A). We did observe dramatically reduced message levels of all tested genes in *atf-7(gf)* and *tir-1(lf)* mutants (Figure 7A, 7B), as previously reported [30], [36].

Our data thus far indicated that DAF-19 is required for TIR-1 signaling to upregulate *tph-1* in the ADF neurons and candidate antimicrobial genes in the intestine. We wished to determine whether DAF-19 regulates every TIR-1 target, or it selectively mediates TIR-1 regulation of pathogen inducible genes. It has been well established that, TIR-1 specifies asymmetrical expression of the olfactory receptor STR-2 in one of two AWC olfactory neurons during the development [23]. We observed that neither AWC neuron expressed *str-2::gfp* in the *tir-1(yz68gf)* mutant, as seen in mutants with excessive TIR-1 activity [23]. However, *daf-19* mutants did not exhibit the AWC phenotype seen in *tir-1(lf)* mutants (Figure S4). Thus, DAF-19 is critical for TIR-1 signaling to induce transcriptional responses to pathogenic bacteria in the 5-HT neurons and the intestine, but is not required for TIR-1 to regulate neural development.

### PA14 triggers TIR-1 signaling via tissue-specific mechanisms

**Figure 8:**
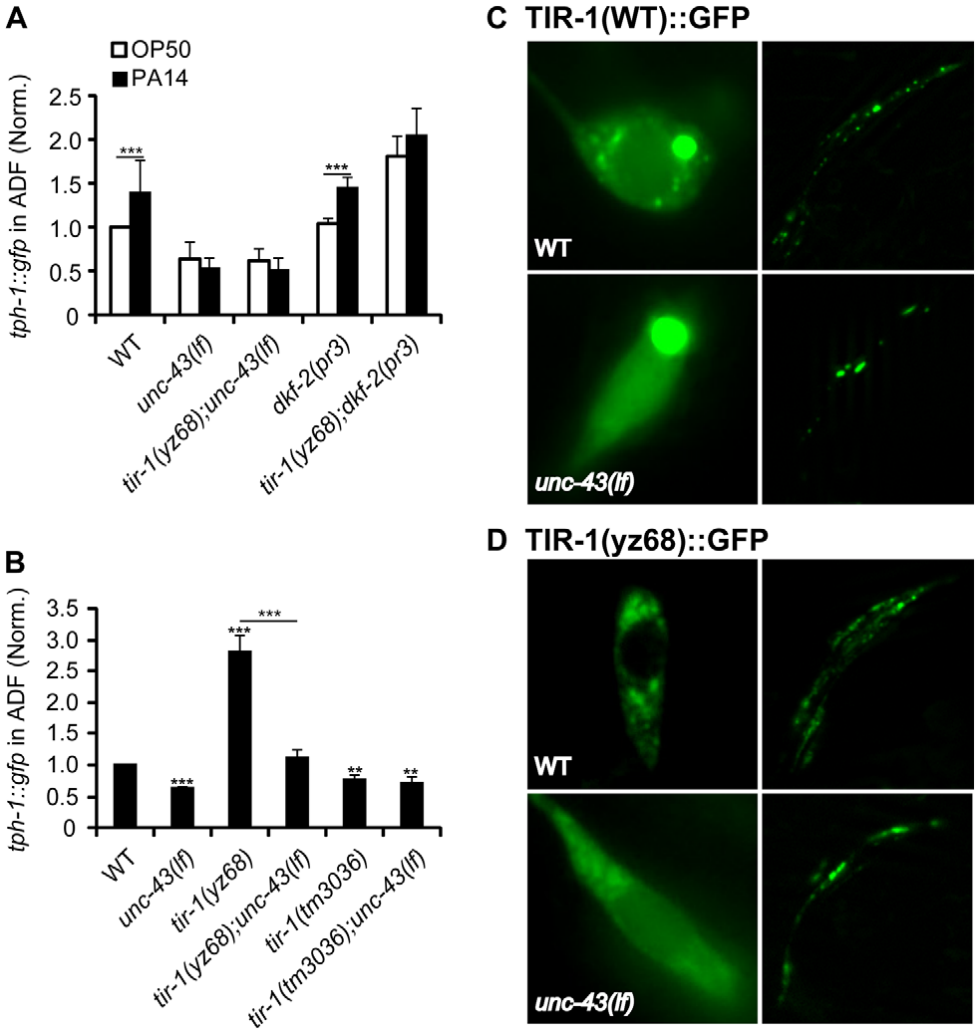
Regulation of TIR-1 signaling in ADF neurons. A. *tph-1::gfp* in the ADF neurons of CaMKII *unc-43(lf)* and PKD *dkf-2(lf) mutants* fed PA14 for 6 hr compared with corresponding sibling fed OP50. *dkf-2* deficiency cannot block *tph-1::gfp* upregulation by PA14 or *tir-1(yz68gf)*. *unc-43(lf)* mutants failed to upregulate ADF *tph-1::gfp* following PA14 treatment. B. *tph-1::gfp* in the ADF neurons of *unc-43(lf)* mutants compared with that in *tir-1(lf)* and *tir-1(gf)* mutants under optimal growth conditions. *unc-43(lf)* suppressed increased *tph-1::gfp* expression by *tir-1(yz68gf)*. Data represent the average of three trials each with at least 15 animals per strain ± SEM. The value of GFP fluorescence in WT animals fed PA14 and that of mutants fed OP50 or PA14 is normalized to the value of WT animals fed OP50. Statistics between WT and individual mutants is marked on the top of each bar, and that between two indicated groups is marked on the top of lines, ** p<0.01, *** p<0.001. C. Representative images of TIR-1(WT)::GFP driven by the *gpa-13* promoter in ADF, ASH and AWC neurons in WT and *unc-43(lf)* backgrounds. The left panels show the cell bodies of ADF neurons, and the right panels show the axons of the neurons around the nerve ring. In *unc-43(lf)* background, the number of punctate structures in the ADF cell body was reduced. The number of puncta in the axons was also reduced although the changes appeared to be less pronounced. D. Images of TIR-1(yz68)::GFP driven by the *gpa-13* promoter in WT and *unc-43(lf)* backgrounds. In WT background, TIR-1(yz68)::GFP showed increased punctate structures as compared to TIR-1(WT)::GFP. In *unc-43(lf)* background TIR-1(yz68)::GFP punctate structures were diminished and the fluorescence in the cell bodies became diffuse.

The finding of the shared transcriptional effectors of TIR-1 signaling in the ADF neurons and intestine raised an intriguing question as to whether pathogenic bacterial signals trigger the neurons and immune cells in the same manner in *C. elegans*. Although little is known about how the worm senses the presence of pathogens and relays the signals to TIR-1, the protein kinase D DKF-2 is thought to promote transcriptional responses to PA14 by activating the TIR-1 signaling pathway [37]. However, we found that the *dkf-2(pr3)*-null mutation did not suppress *tph-1::gfp* upregulation by *tir-1(yz68gf)* or PA14 (Figure 8A).

Previously, we showed that a gain-of-function mutation in the calcium/calmodulin-dependent protein kinase II (CaMKII) UNC-43 upregulates ADF *tph-1::gfp* [4]. We hypothesized that UNC-43 could be a component of the TIR-1 signaling pathway in the ADF neurons, similar to its involvement in TIR-1-mediated AWC development [23]. To test this hypothesis, we analyzed *tph-1::gfp* in *unc-43(lf);tir-1(yz68)* double mutant. We observed that *unc-43(lf)* abrogated ADF *tph-1::gfp* upregulation in *tir-1(yz68)* mutants (Figure 8B). Furthermore, PA14 failed to induce ADF *tph-1::gfp* upregulation in both the *unc-43(lf)* single and *unc-43(lf);tir-1(yz68gf)* double mutants (Figure 8A). We previously showed that *unc-43(lf)* does not block ADF *tph-1::gfp* upregulation during dauer formation [5]. These observations together suggest that UNC-43 selectively regulates TIR-1-mediated *tph-1* upregulation and that *tir-1(yz68gf)* cannot bypass UNC-43 function.

In cultured mammalian neurons, CaMKII activation alters the subcellular localization of signaling components to initiate cellular responses [38]. UNC-43 has been shown enriched in postsynaptic sites of a number of neuronal types and was co-immunopecipitated with TIR-1 when co-expressed in cultured mammalian cells [23], [39]. If UNC-43 regulates TIR-1 subcellular distribution, then it is possible that the subcellular distribution of the TIR-1(yz68gf) protein also depends on UNC-43. We tested this idea by comparing the subcellular distribution of GFP-tagged TIR-1 and TIR-1(yz68gf) proteins expressed in chemosensory neurons by the *gpa-13* promoter in WT and *unc-43(lf)* backgrounds. In WT animals, TIR-1(WT)::GFP was observed in punctate structures in the axons around nerve ring, with more diffused fluorescence seen in the cell bodies (Figure 8C). By contrast, TIR-1(yz68gf)::GFP displayed increased punctate structures in the axons as well as in the cell bodies (Figure 8D). Importantly, TIR-1(WT)::GFP and TIR-1(yz68gf)::GFP punctate structures were reduced in *unc-43(lf)* mutants (Figure 8C, 8D). Based on these observations, we speculate that the C426Y substitution of TIR-1(yz68gf) alters the protein conformation, thereby enhancing its interaction with UNC-43 in particular cellular compartments where TIR-1 interacts with MAPK signaling components, but that TIR-1(yz68gf) cannot efficiently interact with its downstream components in the absence of UNC-43. Collectively, these results suggest that PA14 triggers distinct mechanisms to activate TIR-1 signaling to induce DAF-19/ATF-7 targets in the ADF neurons and the intestine. Our data indicate that UNC-43 is required for TIR-1 signaling in ADF, and DKF-2 is not.

## Discussion

**Figure 9:**
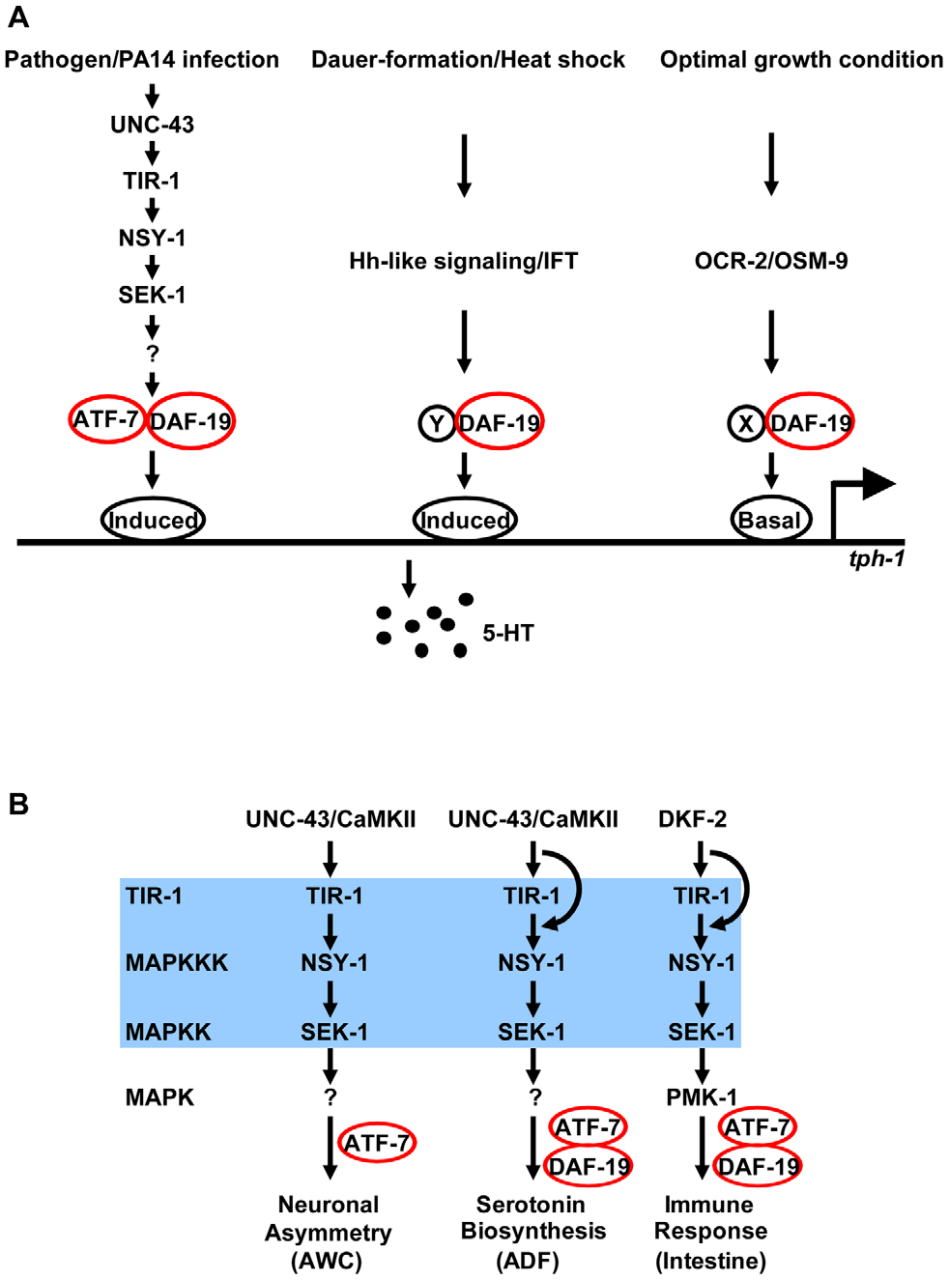
DAF-19 regulation of 5-HT biosynthesis and innate immunity. A. DAF-19 is engaged in multiple independent signaling pathways that regulate *tph-1* expression in ADF neurons. Based on the genetic interaction of DAF-19 and ATF-7 in the TIR-1 signaling pathway, we speculate that DAF-19 interacts with different cofactors at different cis-elements of the *tph-1* promoter in response to different environmental cues. DAF-19 may simultaneously interact with multiple cofactors to confer additive upregulation of *tph-1* in response to multiple aversive cues. B. DAF-19 regulates TIR-1 targets in response to pathogenic bacterial infection, but is not required for TIR-1 to regulate AWC neuron development. However, pathogenic bacterial infection may trigger the TIR-1-DAF-19/ATF-7 pathway via cell-specific mechanisms.

Here, we used an unbiased genetic approach to identify molecular mechanisms underlying transcriptional responses to pathogenic bacterial infection in *C. elegans*. We identified DAF-19, the ortholog of RFX transcription factors. We showed that DAF-19 acts downstream in the TIR-1 pathway to induce *tph-1* in the ADF neurons and antimicrobial genes in the intestine. Our genetic analyses suggest that bacterial infection may trigger the TIR-1 signaling cascade via cell-specific mechanisms, but common TIR-1 downstream transcription factors regulate neuronal and immune responses to infection. Our data demonstrat that RFX gene function, which is required for human adaptive immunity, regulates 5-HT biosynthesis and innate immunity in *C. elegans* (Figure 9).

### DAF-19 is an ancient transcriptional regulator of innate immunity

Parallel to the *C. elegans* NSY-1/MAPKKK-SEK-1/MAPKK-PMK-1/p38 MAPK pathway, the ASK-1/MAPKKK to p38 MAPK pathway regulates mouse innate immunity [40]. While members of the NF-κB family of transcription factors are the major effectors in mammalian innate immune responses, the ASK-1-p38 innate immune pathway is independent of NF-κB [40]. Since *C. elegans* lacks NF-κB, this MAPK pathway was proposed to act via effectors evolutionarily more ancient than NF-κB in the host defense systems [40]. In this study, we identify that DAF-19 RFX is a transcription factor downstream of this core innate immune pathway in *C. elegans*. Our genetic analyses suggest that DAF-19 concerts with ATF-7, a member of the ATF/CREB superfamily of transcription factors, to upregulate *tph-1* in the ADF neurons and antimicrobial factors in the intestine in response to the pathogenic *P. aeruginosa* strain PA14, reminiscent of the RFX-CREB partnership for human MHC II gene expression.

Several lines of evidence point to RFX factors as an ancient mechanism for enhancing survival under aversive conditions, one predating the divergence of stress responses and immunity. First, RFX is a regulator of cell cycle in a nutrient sensing pathway in *Schizosaccharomyces pombe* [41] and an effector of the DNA damage and replication checkpoint pathway essential for *Saccharomyces cerevisiae* survival under replicative stress [42]. Second, biochemical experiments have identified a role for RFX factors in RAS signaling-regulated transcription in mammalian epithelial cells [43]. Third, in addition to mediate transcriptional responses to PA14, *daf-19* controls the decision to enter the stress-resistant dauer stage that is specialized for enduring aversive environmental conditions [17]. Fourth, 5-HT is perhaps one of the most ancient mechanisms of stress responses conserved across phyla [44]. Pharmacological and biophysical experiments have long demonstrated that 5-HT biosynthesis in mammals is highly sensitive to environmental conditions and can be upregulated by a variety of metabolic, psychological and physical stressors in a region-specific manner [45], [46], although systematic dissection of 5-HT biosynthesis in the mammalian brain has not been feasible. Our results raise the possibility that the mechanisms that regulate 5-HT biosynthesis and certain aspects of innate immunity may be interrelated.

What could be the mechanism by which DAF-19 regulates *tph-1* and those antimicrobial genes? Earlier studies of RFX regulation of human MHC II genes provided evidence that RFX factors regulate gene expression indirectly through recruiting additional transcriptional regulators to the promoters [13]. In a recent study, RFX factors were shown to protect promoters against epigenetic silencing by DNA methylation through recruiting chromatin-remodeling factors [47]. Thus one plausible mechanism could be that DAF-19 and ATF-7 bind to a promoter element shared in common among the pathogen-inducible genes, and infection induces the TIR-1 signaling cascade leading to phosphorylation of ATF-7 [30] and transcriptional activation of the targets. However, genomewide X-box motif search identified hundreds of candidate genes, only a few antimicrobial genes are among them [48] and no X-box can be recognized in the promoter of the *tph-1*, *T24B8.5*, *C17H12.8*, *K08D8.5 or F35E12.5* genes. Thus, DAF-19 is likely to bind non-consensus X-boxes on the promoters via co-regulators. Alternatively, DAF-19 may modulate chromatin structure, facilitating the binding of other transcriptional regulators to the promoters. We favor the binding-via-cofactor model because our data thus far suggest that DAF-19 is involved in multiple environment-dependent regulations of *tph-1* expression, whereas ATF-7 selectively regulates the response to PA14. We speculate that DAF-19 interacts with distinct cofactors that are regulated by distinct environmental cues (Figure 9A). Further experiments are required to determine whether DAF-19 directly interacts with ATF-7. In addition, the possibility that DAF-19 and ATF-7 regulate yet unidentified transcription factor(s), which in turn regulate pathogen-inducible genes, cannot be excluded. It is perhaps interesting to note that our two *daf-19* alleles both are located in the predicted dimerization (DIM) domain, suggestive for the importance of protein-protein interaction in DAF-19 function.

While the exact mechanism of DAF-19 on the target gene promoters remains to be elucidated, our data showed that DAF-19 and ATF-7 regulate common immune gene markers. Remarkably, by comparing the list of TIR-1 signaling targets identified by microarray analysis [36] with the database of several independent expression profiling of *daf-19* mutants [49], [50], [51], we found that 102 out of 215 TIR-1 gene targets are among those differentially expressed in *daf-19* mutants; one of them is *C17H12.8*, which was confirmed by our qPCR. We showed that *daf-19* mutations can suppress increased expression of immune gene markers in *tir-1(yz68gf)* mutants. However, *daf-19*-null;*tir-1(yz68gf)* double mutants did not display reduced expression of those immune genes as seen in the *daf-19* single mutants, judging by *T24B8.5::gfp* in living worms and qPCR. Consistent with the gene expression analyses, the *daf-19*-null mutation blocked the enhanced resistance to killing by PA14 in *tir-1(yz68gf)* mutant but the *daf-19;tir-1(yz68gf)* double mutant did not exhibit heightened susceptibility as *daf-19* single mutants did. These observations may be consistent with the model in which DAF-19 and ATF-7 interact with additional transcription factor(s) that also contribute to the regulation of those antimicrobial genes.

### Multiple mechanisms relay pathogen signals to the TIR-1-DAF-19 signaling pathway

There is converging evidence from neurobiology and immunology suggesting that the brain immune privilege is not absolute [52]. Internal and external pathogenic products can infiltrate into the CNS and there is extensive bi-directional communications between the CNS and immune systems [53], [54]. The finding of a large number of classical immune proteins in the CNS has led to the idea that common molecular mechanisms may be involved in neuronal and immune responses to pathogenic signals [1], [2], [53]. Our identification of the TIR-1-DAF-19/ATF-7 pathway in regulating *tph-1* and antimicrobial genes supports this idea. Moreover, our genetic analysis suggests that activation mechanisms of this core signaling pathway in the neurons and intestine during infection differ. A prior work showed that DKF-2 is required for TIR-1 signaling cascade to induce intestinal immunity [37]. We found that *dkf-2*-null did not prevent PA14-induced *tph-1::gfp* upregulation. Instead, we identified a requirement of UNC-43 CaMKII for *tph-1::gfp* upregulation induced by PA14 and *tir-1(yz68gf)*. Analysis of TIR-1(WT)::GFP and TIR-1(yz68)::GFP suggests that UNC-43 regulates subcellular distribution of TIR-1. While several mammalian TIR domain adaptor proteins have been shown to translocate to the plasma membrane following Toll-like receptor activation, SARM, the ortholog of TIR-1, is activated in the brain by neural toxicity via a yet unidentified mechanism [55], [56]. Our results raise the possibility that the cue associated with pathogenic bacterial infection triggers Ca^2+^ signaling to activate TIR-1 in the ADF neurons. It may be sensible that neurons and immune cells detect distinct molecular cues associated with infection thereby coordinating neuronal and physiological responses.

## Materials and Methods

### Strains

*C. elegans* strains were maintained at 20°C on NGM agar plates seeded with a lawn of *Escherichia coli* OP50 as a food source. WT animals were Bristol strain *N2*. The Hawaiian isolate *CB4856* was used in genetic mapping of the *daf-19* and *tir-1* mutations. The following existing mutant strains were used in this study: *atf-7(qd22gf)*, *atf-7(qd22 qd130lf)*, *daf-6(e1377)*, *daf-19(m86)*, *dkf-2(pr3)*, *dyf-1(yz66)*, *eri-1(mg366);lin-15B(n744)*, *ocr-2(yz5)*, *sek-1(km4)*, *tir-1(ok1052)*, *tir-1(tm3036lf)*, *unc-43(e408)*. Transgenic strains used in this study were: *agIs219*: *Is[T24B8.5::gfp; ttx-3::gfp]* [30], *CX3695: kyIs140[str-2::gfp; lin-15(+)]* [57], *GR1333: yzIs71[tph-1::gfp; Rol-6(d)]* [*21*], *Is[cat-1::gfp]* [*58*], *JY222: ExZ042[ocr-2::gfp; Rol-6(d)]* [4], *JY449: ExX002[str-2::gfp; Rol-6(d)]* [*59*].

### Identification of *tir-1(yz68)*, *daf-19(yz69)*, and *daf-19(yz70)* mutations

*yz68* is a dominant mutation isolated from a genetic screen for mutants with enhanced GFP expression in ADF chemosensory neurons after ethyl methane sulfonate (EMS) mutagenesis of *ocr-2(yz5)* mutant carrying an integrated *tph-1::gfp* transgene as described previously [5]. Genetic mapping using single-nucleotide polymorphisms (SNP) of *CB4856* localized *yz68* to a contig of 1.43 map on the chromosome III. To identify the mutant gene, 174 genes located in the contig were individually inactivated in *yz68* mutants by RNAi, and the clone F13B10.1 expressing double stranded(ds)-RNA of *tir-1* suppressed the *tph-1::gfp* upregulation of the *yz68* mutant. Sequencing *yz68* genomic DNA revealed a G to A transition resulting in a cysteine_426_ to tyrosine substitution in the fourth ARM motif of the HEAT/Arm repeat region of TIR-1. The amino acid altered in *yz68* is in reference of the *tir-1a* isoform.

*yz69* and *yz70* mutants are recessive mutations isolated from an EMS mutagenesis screen for mutants with dramatically reduced/absence of ADF *tph-1::gfp*. Analysis of the amphid morphology with fluorescence dye DiI revealed that ciliated neurons in *yz69* and *yz70* mutants were dye filling defective. *CB4856* SNP mapping localized *yz69* to a contig of 1.28 map units between the polymorphisms in the clones C18D1 and F44F4 on the chromosome II. Non-complementation assays with dye-filling mutants within the region indicated that both *yz69* and *yz70* were allelic to *daf-19*. Sequencing the *daf-19* gene of the mutants revealed in *yz69* a G to A transition predicting a cysteine to tyrosine substitution at the conserved dimerization domain, and in *yz70* a G to A transition predicting an opal mutation in the dimerization domain. The amino acid changes depicted in Figure 3A are in reference of *daf-19a* isoform.

### Generation of transgenic animals

All constructs were generated by PCR. *daf-19(g)* was a ∼14.8 kb genomic fragment amplified from the WT genome encompassing 2.9 kb 5′-upstream promoter sequence, exons/introns, and 574 bp 3′-UTR of the *daf-19* gene.

To express *tir-1* and *daf-19* in specific neurons, we fused *tir-1* and *daf-19* cDNA sequences individually to the *gpa-13* promoter, which is expressed in three pairs of amphidal ciliated sensory neurons ADF, ASH and AWC. The *gpa-13* promoter is expressed additionally in PHA and PHB phasmid neurons located in the tail [60]. *tir-1(WT)* and *tir-1(yz68)* cDNA were amplified from cDNA mixture prepared from total RNA of WT and *tir-1(yz68)* animals, respectively, using primers corresponding to the *tir-1a* isoform. The cDNA of the *daf-19c* isoform was amplified from the plasmid PS0243 (kindly provided by P. Swoboda). The 2.6 kb *gpa-13* promoter sequence amplified from the plasmid PS0243 was fused to the sequences in the order of a cDNA, GFP and *unc-54* 3′UTR or a cDNA, mCherry and *unc-54* 3′UTR. To express DAF-19 in the intestine, the cDNA sequence of *daf-19a* isoform was fused to the 2.9 kb *ges-1* promoter. For each construct, products from three independent PCR reactions were pooled to reduce potential PCR errors. The pooled PCR products were purified (Qiagen) and microinjected at the concentration of 50 ng/µl into worms. The plasmid containing either a dominant *rol-6* gene (*Rol-6(d)*), *elt-2::gfp* or *unc-122::RFP* was co-injected as a transgenic marker.

### RNAi

All RNA interference (RNAi) experiments were done in the background of *eri-1;lin-15B*, which enhances sensitivity to RNA interference in neurons [61]. RNAi assays were carried out by feeding worms *E. coli HT1115* expressing dsRNA of a target gene or the control empty L4440 vector (Ahringer RNAi library, University of Cambridge, England). RNAi clones were individually cultured overnight in Luria broth containing 100 µg/ml ampicillin, 500 µl of the bacterial culture were seeded onto agar plates containing NGM supplemented with 1 mM IPTG and 25 µg/ml carbenicillin to induce dsRNA expression, and incubated overnight at room temperature. For RNAi of *tir-1* and *nsy-1*, about 60 eggs were placed onto each plate and allowed to hatch, grow to adults and lay eggs. F1 progeny were transferred to a fresh plate, and *tph-1::gfp* in the ADF neurons of L4-stage worms of F2 generation quantified. For developmental RNAi of *daf-19*, synchronized L1 worms were transferred to the plates (day 0), the worms were transferred to freshly prepared plates every day, and the expression of *tph-1::gfp* in ADF neurons or DiI staining of cilia morphology were analyzed on indicated days. DiI staining was done as previously described [5]. For each RNAi experiment, three independent trials each with three replicates were performed, and data from one representative trial presented.

### Indirect immunofluorescence microscopy

Whole-mount staining of worms with anti-5-HT antibody was performed as described previously [21]. The staining patterns were visualized via Alexa Fluor 594 or 488 conjugated secondary antibodies (Molecular Probe) under an AxioImager Z1 microscope equipped with proper filters, and images were captured using AxioCam MR digital camera (Zeiss, Northwood, NY). To quantify the intensity of 5-HT immunoreactivity, images of ADF neurons in individual worms were captured under a 40× objective lens at a fixed exposure time of 3 ms with 100% UV exposure level. For each image, fluorescence intensity of a circular 10 pixels area within the ADF cell body was quantified using the ImageJ software (National Institute of Health, Bethesda, Maryland). To exclude the background, fluorescence intensity over a circular 10 pixels area posterior to the ADF cell body in the same image was quantified, and the value of the background was subtracted from the value of the ADF area.

### Assessment of GFP reporter levels and statistics

The expression of a chromosomally integrated *tph-1::gfp* reporter in ADF neurons in living WT or mutant worms was evaluated by measuring GFP fluorescence intensity. Images of ADF neurons in individual animals were captured at a fixed exposure time. The external contour of each ADF neuron was delineated, and fluorescence intensity within the entire neuron was quantified by using the ImageJ software. L4-stage animals were examined, unless noted otherwise. Ideally mutants that reduce and that upregulate *tph-1::gfp* were assayed in parallel, thus the exposure time was designed to detect reduction as well as upregulation of *tph-1::gfp*. This setting was most reliable at the range of 1–3 fold higher than WT; consequently, we used *ocr-2* mutant background to lower the basal *tph-1::gfp* to evaluate the changes of mutants with constitutively increased ADF *tph-1::gfp*, and the double mutants were compared with *ocr-2* single mutants assayed on the same day.

For quantifying *tph-1::gfp* intensity following PA14 treatment, one-day-old young adult worms were transferred to standard slow killing assay plates [62] seeded with either PA14 or control OP50, incubated at 25°C for 6 hr, images of the ADF neurons were captured and GFP intensity quantified.

For quantifying *tph-1::gfp* intensity in dauers, WT and mutants were induced to form dauers by dauer pheromones. Dauer pheromone and pheromone-containing plates were prepared as we have done previously [5], following an established protocol [63]. 20–25 gravid worms from each strain were transferred to NGM plates supplied with 1 unit of dauer pheromone, allowed to lay eggs for 2–3 hr in a 25°C incubator, the adults were then removed from the plates, and dauers developed from hatched eggs on the plates were analyzed 72 hr later. For each strain the value of dauers was compared to that of L4 grown on the plates without pheromone and assayed on the same day.

The expression of *T24B8.5::gfp* in the intestine was analyzed in two-day-old adult worms carrying the integrated *agIs219[T24B8.5::gfp; ttx-3::gfp]* transgene cultured on OP50 at 20°C. Images of the intestine were captured under a 10× objective lens at a fixed exposure time, and fluorescence intensity was quantified by measuring pixel intensity of three areas along the body of each animal as depicted in Figure 6B.

Data represent the average of at least three trials unless specified otherwise. For each trial, 15–20 animals per strain per condition/treatment were analyzed and compared to the controls assayed on the same day. WT animals under the same conditions and treatments were analyzed for every experiment. Unpaired Student's t-test was used for comparisons between a mutant and WT and between two mutants or two treatments.

### *P. aeruginosa* PA14 pathogenesis assays

The standard PA14 slow killing assays were performed as previously described [62]. Briefly, PA14 was cultured in King's broth overnight and the culture was seeded at the center of 3.5 cm diameter assay plates and incubated at 37°C for 20 hr followed by 20 hr incubation at room temperature. 40–50 L4 worms per strain were transferred onto each assay plate, incubated at 25°C and scored for dead or live every 8 hr. Worms were scored as dead if no response was detected after prodding with a platinum wire. *daf-19* mutants tend to claw off the plate. So for each assay, more than 300 L4 *daf-19* mutants were transferred to each plate, and live and dead animals on the agar surface were scored at indicated time points; dead animals on the wall of the plate not counted. For each strain, three replicates were analyzed for each experiment. To test the effect of RNAi of *daf-19*, 4 to 6 gravid animals were grown on the RNAi plates as described above seed with either *E-coli* HT1115 harboring empty control plasmid L4440 or plasmids expressing RNAi against *daf-19* gene. F1 progeny at the L4 stage were used to test the susceptibility to killing by PA14.

### Quantitative real-time PCR (qPCR) analysis

Total RNA from 100 one-day-old adults of WT and indicated mutant strains was extracted using Trizol (Invitrogen), reserve transcribed to cDNA using the SuperScript III system (Invitrogen), and the cDNA was used for qPCR analyses using the StepOnePlus machine (Applied Biosystems) and SYBR Green detection system (Applied Biosystems) in triplicated reactions. The primers for qPCR were designed using Primer Premier 5.0 (Premier Biosoft) (Figure S5). Values were normalized against the reference gene *act-1* [37]. *gpd-2* was analyzed as a second control showing no change relative to *act-1*. Fold change was calculated using the delta Ct method [64]. qPCR analysis of L4 worms of those strains showed comparable results; data of the adults are presented.

## Supporting Information

Figure S1 Regulation of *tph-1::gfp* expression in the ADF neurons by TIR-1 downstream *nsy-1* MAPKKK and *sek-1* MAPKK RNAi of *nsy-1* suppressed *tph-1::gfp* upregulation by *tir-1(yz68gf)*. RNAi of *nsy-1* and loss-of-function mutation of *sek-1* did not confer *tph-1::gfp* reduction, indicating that the TIR-1 signaling pathway is designated primarily to upregulate *tph-1* expression in response to pathogen infection. Data represent the average of three trials each with at least 15 animals per strain ± SEM. The value of GFP fluorescence in mutants was normalized to that of WT animals, and the value of RNAi-treated animals is normalized to that of mock RNAi with an empty vector. Statistics between WT and individual mutants and RNAi-treated animals is marked on the top of each bar, and that between two indicated groups is marked on the top of lines, *** p<0.001, unpaired student's t test.
(TIF)

Figure S2 *tph-1::gfp* in the ADF neurons of the IFT mutant *dyf-1*. *dyf-1* mutant animals were treated with a mock RNAi with an empty vector or a vector expressing RNAi against *tir-1* or *daf-19*. RNAi of *tir-1* did not reduce *tph-1::gfp* in the ADF neurons, compared to mock RNAi. By contrast, RNAi of *daf-19* abolished ADF *tph-1::gfp*. Data represent the average of three trials each with at least 15 animals per strain ± SEM. The value of RNAi of *tir-1* and *daf-19* is normalized to that of *dyf-1* mutants treated with the empty vector. *** p<0.001, unpaired student's t test.
(TIF)

Figure S3 *daf-19* is expressed in the intestine. Top, an image of L4 worm expressing GFP driven by a genomic fragment encompassing 2.9 kb 5′-upstream sequence to exon 8 of *daf-19*. Bottom, a bright field image showing the position of the same worm. Anterior is to the left, and arrows point to the nuclei of the intestinal cells.
(TIF)

Figure S4 DAF-19 is not required for TIR-1 regulation of AWC cell fates, while *atf-7* is. A–D. Photomicrographs showing L4 animals expressing an integrated GFP reporter for the olfactory receptor *str-2* (*str-2::gfp*). *daf-19* and WT animals expressed *str-2::gfp* stochastically in one of two AWC neurons. Neither AWC expressed *str-2::gfp* in *tir-1(yz68gf)* mutants. E. Quantification of *str-2::gfp* expression in AWC neurons in *daf-19*, *atf-7* and *tir-1* signaling mutants. *atf-7(gf)* mutants exhibited *str-2::gfp* expression pattern as seen in *tir-1(lf)* mutants, but *atf-7(lf)* showed mixed *str-2::gfp* patterns of *tir-1(lf)* and *tir-1(yz68gf)*. *daf-19* did not display the *str-2::gfp* phenotype of *tir-1(lf)*, nor suppressed the *str-2::gfp* phenotype of *tir-1(yz68gf)*. Data represent the percentage of the animals of a strain showing each AWC phenotype. n, number of animals analyzed.
(TIF)

Figure S5 Primer sequences used for qPCR analysis of candidate antimicrobial genes.
(TIF)
