# Supplementary figures and images for: RFX Transcription Factor DAF-19 Regulates 5-HT and Innate Immune Responses to Pathogenic Bacteria in Caenorhabditis elegans
Source: PLoS Genet. 2013 Mar 7;9(3):e1003324. doi: 10.1371/journal.pgen.1003324 (PMC3591283; doi:10.1371/journal.pgen.1003324)

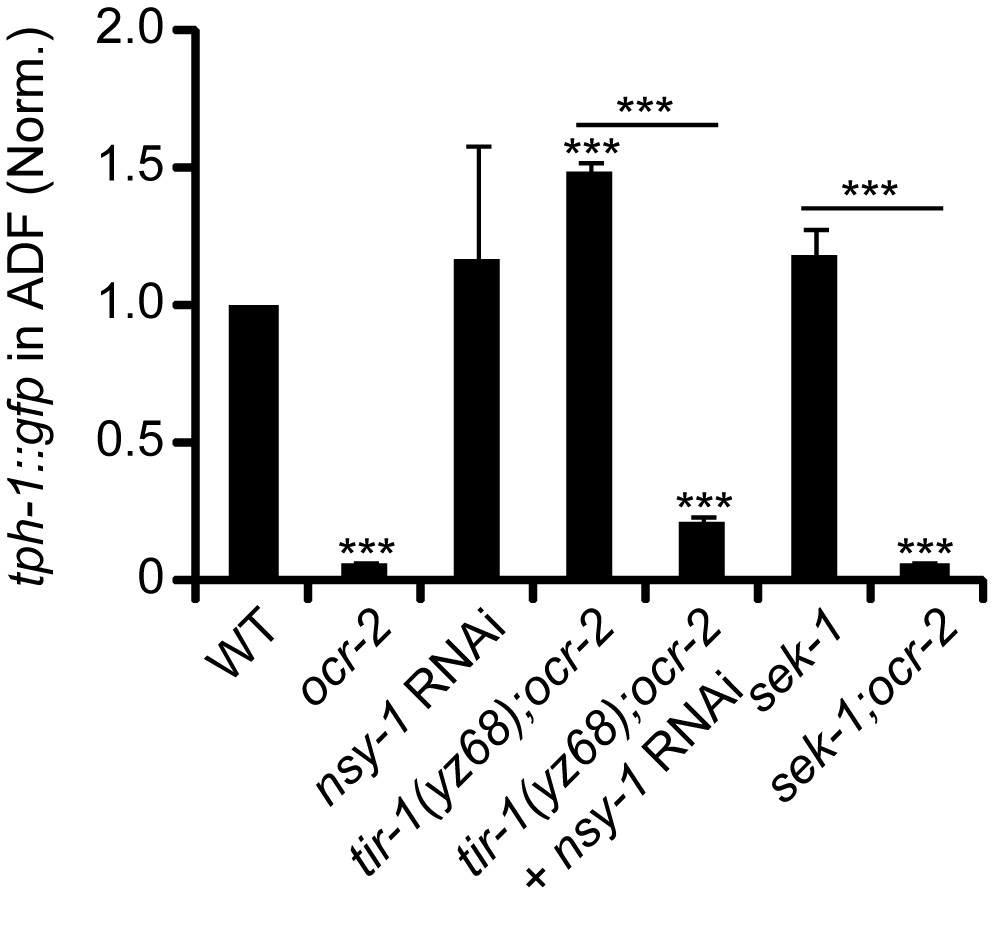

Supplement: Figure S1 — Regulation of tph-1::gfp expression in the ADF neurons by TIR-1 downstream nsy-1 MAPKKK and sek-1 MAPKK RNAi of nsy-1 suppressed tph-1::gfp upregulation by tir-1(yz68gf). RNAi of nsy-1 and loss-of-function mutation of sek-1 did not confer tph-1::gfp reduction, indicating that the TIR-1 signaling pathway is designated primarily to upregulate tph-1 expression in response to pathogen infection. Data represent the average of three trials each with at least 15 animals per strain ± SEM. The value of GFP fluorescence in mutants was normalized to that of WT animals, and the value of RNAi-treated animals is normalized to that of mock RNAi with an empty vector. Statistics between WT and individual mutants and RNAi-treated animals is marked on the top of each bar, and that between two indicated groups is marked on the top of lines, *** p<0.001, unpaired student's t test. (TIF) [file pgen.1003324.s001.tif]

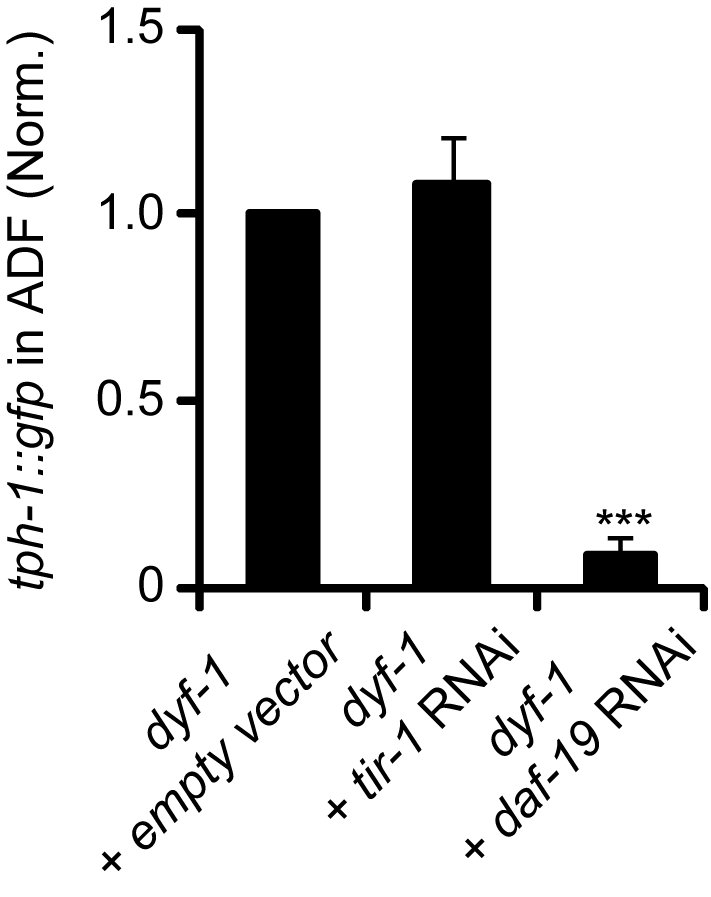

Supplement: Figure S2 — tph-1::gfp in the ADF neurons of the IFT mutant dyf-1. dyf-1 mutant animals were treated with a mock RNAi with an empty vector or a vector expressing RNAi against tir-1 or daf-19. RNAi of tir-1 did not reduce tph-1::gfp in the ADF neurons, compared to mock RNAi. By contrast, RNAi of daf-19 abolished ADF tph-1::gfp. Data represent the average of three trials each with at least 15 animals per strain ± SEM. The value of RNAi of tir-1 and daf-19 is normalized to that of dyf-1 mutants treated with the empty vector. *** p<0.001, unpaired student's t test. (TIF) [file pgen.1003324.s002.tif]

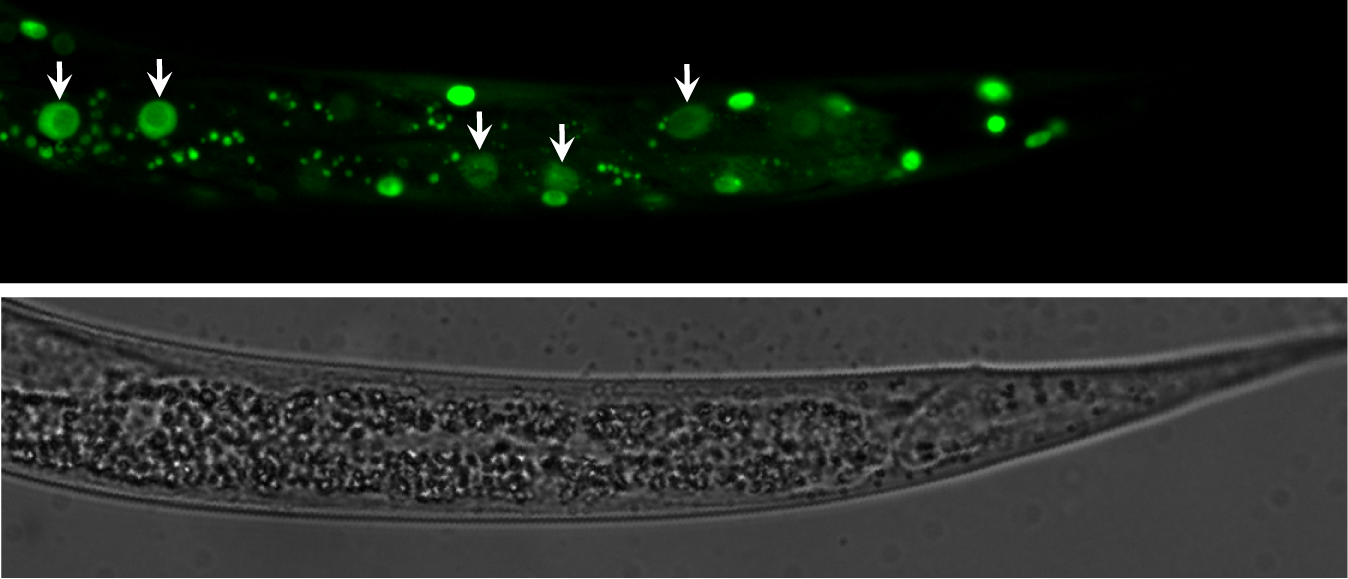

Supplement: Figure S3 — daf-19 is expressed in the intestine. Top, an image of L4 worm expressing GFP driven by a genomic fragment encompassing 2.9 kb 5′-upstream sequence to exon 8 of daf-19. Bottom, a bright field image showing the position of the same worm. Anterior is to the left, and arrows point to the nuclei of the intestinal cells. (TIF) [file pgen.1003324.s003.tif]

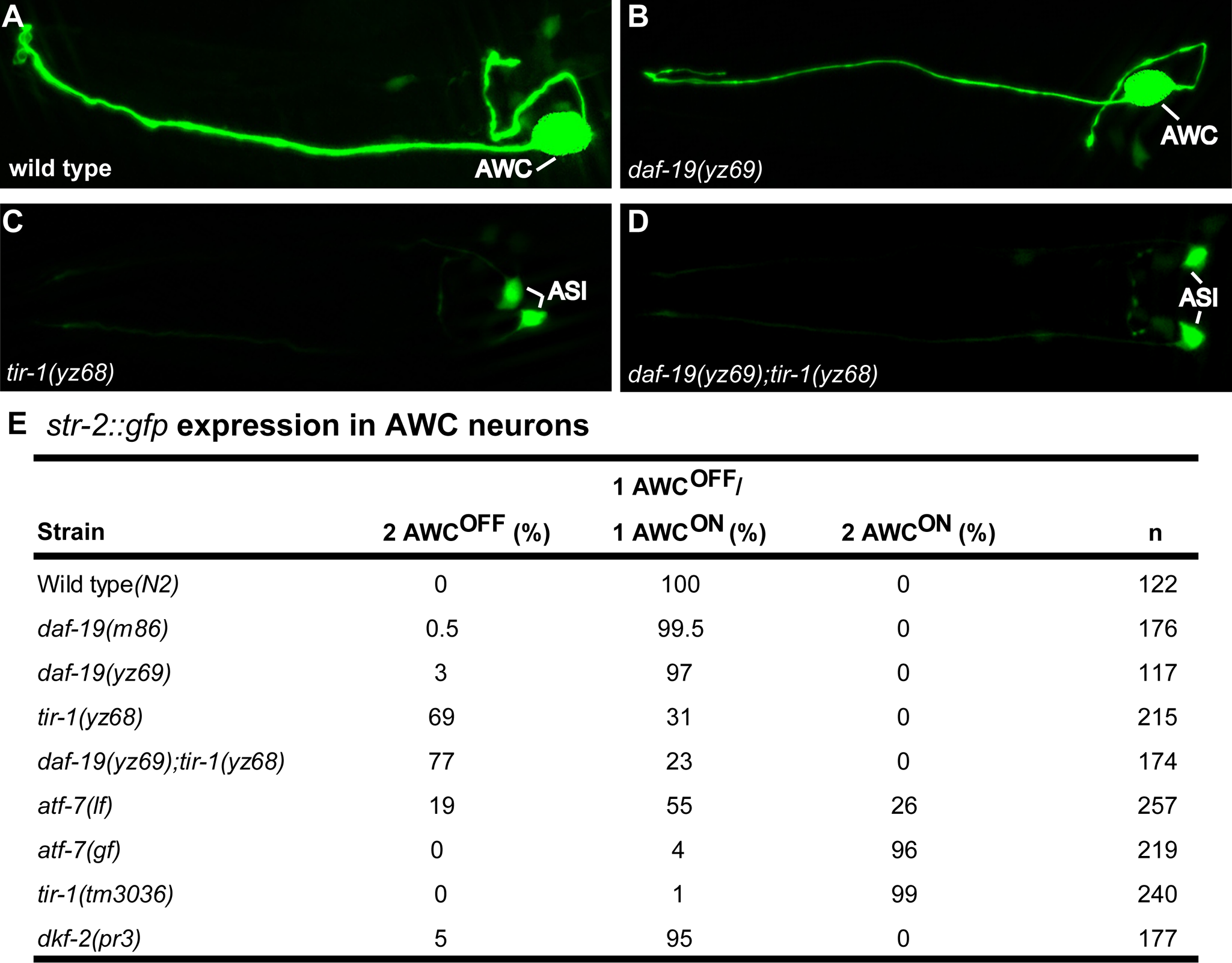

Supplement: Figure S4 — DAF-19 is not required for TIR-1 regulation of AWC cell fates, while atf-7 is. A–D. Photomicrographs showing L4 animals expressing an integrated GFP reporter for the olfactory receptor str-2 (str-2::gfp). daf-19 and WT animals expressed str-2::gfp stochastically in one of two AWC neurons. Neither AWC expressed str-2::gfp in tir-1(yz68gf) mutants. E. Quantification of str-2::gfp expression in AWC neurons in daf-19, atf-7 and tir-1 signaling mutants. atf-7(gf) mutants exhibited str-2::gfp expression pattern as seen in tir-1(lf) mutants, but atf-7(lf) showed mixed str-2::gfp patterns of tir-1(lf) and tir-1(yz68gf). daf-19 did not display the str-2::gfp phenotype of tir-1(lf), nor suppressed the str-2::gfp phenotype of tir-1(yz68gf). Data represent the percentage of the animals of a strain showing each AWC phenotype. n, number of animals analyzed. (TIF) [file pgen.1003324.s004.tif]

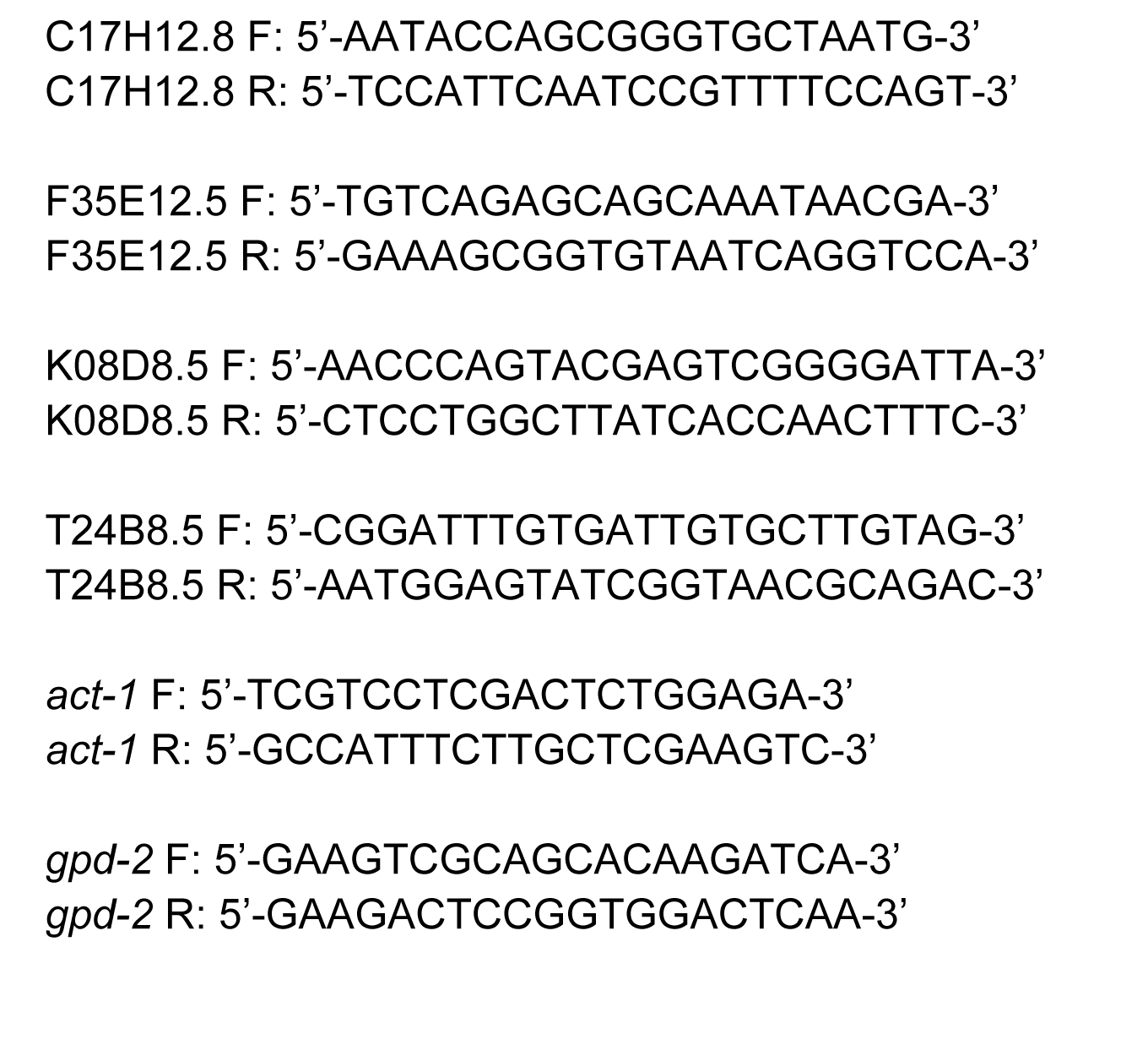

Supplement: Figure S5 — Primer sequences used for qPCR analysis of candidate antimicrobial genes. (TIF) [file pgen.1003324.s005.tif]
